# ROS‐Targeted Nanomotor Therapy in OA: Cartilage Protection and Pain Relief

**DOI:** 10.1002/advs.76314

**Published:** 2026-07-06

**Authors:** Meng Zheng, Changyu Liu, Qin Xia, Arndt F. Schilling, Jiawei Jiang, Renpeng Peng, Zixing Shu, Tian Ma, Danni Luo, Yaoyu Zhang, Yibo Fan, Xuyuan Zhang, Song Li, Kai Wang, Wentao Ke, Yuan Xiong, Yuanli Zhu, Fangzhi Mou, Jun Xiao, Hao Zhu

**Affiliations:** ^1^ Department of Orthopaedics Tongji Hospital Tongji Medical College Huazhong University of Science and Technology Wuhan China; ^2^ Department of Trauma Surgery Orthopaedics and Plastic Surgery University Medical Center Göttingen Göttingen Germany; ^3^ Department of Pathology Tongji Hospital Tongji Medical College Huazhong University of Science and Technology Wuhan China; ^4^ State Key Laboratory of Advanced Technology for Materials Synthesis and Processing Wuhan University of Technology Wuhan China

**Keywords:** metformin, nanomotor, NRF2, osteoarthritis, pain

## Abstract

Osteoarthritis (OA) is a prevalent degenerative joint disease with limited effective treatment options. Joint inflammatory pain is a primary reason patients seek care, but systemic drug administration often causes severe side effects, while intra‐articular injection suffers from rapid clearance and poor tissue penetration. Herein, we develop nanozyme‐based Janus nanomotors loaded with metformin (MET) for reactive oxygen species (ROS)‐targeted therapy in OA. Leveraging intrinsic superoxide dismutase (SOD) and catalase (CAT) enzymatic activity, these nanomotors harness pathologically elevated ROS within the articular microenvironment as chemical fuel, achieving self‐propelled deep penetration into both cartilage and synovial tissue. This mechanism facilitates simultaneous on‐the‐move ROS scavenging and sustained deep‐tissue MET release, which further restores redox homeostasis and protects chondrocytes by regulating the NRF2/KEAP1 signaling pathway. Furthermore, the nanomotors can suppress nociceptive signaling in the dorsal root ganglia (DRG), thereby alleviating joint pain and improving mobility. This research offers a novel and efficient approach for cartilage protection and arthritis pain management.

## Graphical abstract

ROS‐responsive NM@MET nanomotors utilize elevated ROS in osteoarthritic joints to promote deep penetration into cartilage and synovium and enable sustained metformin release. By scavenging ROS, regulating NRF2/KEAP1 signaling, protecting chondrocytes, suppressing synovial inflammation, and attenuating nociceptive signaling, NM@MET alleviates pain and improves joint function.

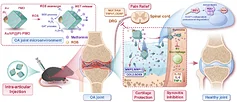

## 1 Introduction

Although OA has long been viewed as a purely “wear‐and‐tear” disorder, mounting evidence shows that low‐grade, chronic inflammation is a key driver of its onset and progression [1]. Mechanical stress and micro‐injuries in the joint activate innate immune pathways in synovium, cartilage, and subchondral bone [2]. This process leads to the production of pro‐inflammatory cytokines such as IL‐1β, TNF‐α, and IL‐6 that sensitize nociceptors [3], along with prostaglandins and bradykinin that directly stimulate pain receptors in the synovium, cartilage, and subchondral bone [4]. Additionally, inflammation leads to synovial thickening, joint effusion, and increased intra‐articular pressure [5]. Despite widespread use, current treatments targeting inflammation in OA have notable limitations and potential downsides. Long‐term use of NSAIDs can lead to gastrointestinal issues, including ulcers and bleeding, as well as increased risks of cardiovascular and kidney problems, particularly in older adults [6]. Corticosteroid injections, while effective for short‐term pain relief, may contribute to cartilage degradation and joint instability if used repeatedly or at high doses [7]. Biologic therapies and cytokine inhibitors, though promising, are expensive, require close monitoring, and carry risks of immune suppression and infection [8]. Moreover, the overall efficacy of these treatments in halting OA progression remains modest, as they often address symptoms rather than the complex, multifactorial nature of the disease. As a result, there remains a critical need for more targeted, disease‐modifying strategies that provide effective pain management while maintaining favorable safety profiles.

Critically, the inflammatory mediators upregulate damage‐associated molecular patterns (DAMPs) and reactive oxygen species (ROS) [9, 10]. In OA‐affected joints, elevated levels of ROS, including superoxide anions, hydrogen peroxide, and hydroxyl radicals, are produced by chondrocytes, synoviocytes, and immune cells [11, 12]. These reactive molecules damage cellular components such as DNA, lipids, and proteins, impairing chondrocyte viability and function [13]. ROS also activate matrix metalloproteinases (MMPs) and aggrecanases, enzymes that break down key components of the cartilage extracellular matrix [14]. Moreover, ROS amplify inflammatory signaling pathways like NF‐κB, creating a vicious cycle that sustains low‐grade inflammation and accelerates joint degeneration [15]. This oxidative stress not only exacerbates cartilage loss but also contributes to synovial inflammation and subchondral bone changes, closely linked to pain through both peripheral and central mechanisms [16]. Therefore, reducing intra‐articular ROS to mitigate local inflammation is anticipated to raise the activation threshold of nociceptors, thus reducing pain. Collectively, these findings highlight ROS as a potential therapeutic target in the management of OA [17, 18].

Metformin (MET) has emerged as an effective ROS‐targeted agent that safeguards cartilage and alleviates pain. It decreases ROS primarily through activation of AMP‐activated protein kinase (AMPK), which plays a central role in cellular energy balance and oxidative stress regulation [19, 20]. By activating AMPK, metformin enhances mitochondrial efficiency and inhibits complex I of the mitochondrial respiratory chain, leading to reduced electron leakage and lower ROS production [21]. Additionally, metformin upregulates antioxidant defenses by increasing the expression of enzymes such as superoxide dismutase (SOD) and catalase, which neutralize ROS [22]. It also suppresses the activity of NADPH oxidase, a major enzyme complex responsible for ROS generation in inflammatory cells [23]. Through these mechanisms, metformin not only reduces oxidative damage but also interrupts the feedback loop between ROS and inflammatory signaling pathways, such as NF‐κB, thereby contributing to its anti‐inflammatory and potentially chondroprotective effects in osteoarthritis. A randomized controlled clinical trial demonstrated that metformin can considerably reduce pain symptoms in overweight or obese patients with knee osteoarthritis [24].

However, the efficacy of MET for OA is still hindered by delivery challenges. Systemic administration lacks joint specificity, while local intra‐articular injection suffers from rapid clearance [25]. Furthermore, cartilage is a dense, avascular, and negatively charged matrix that acts as a formidable physical barrier to passive drug diffusion [26, 27]. Consequently, conventional carriers fail to penetrate deep into the affected tissue, treating only the superficial layers and necessitating frequent, invasive reinjections [28]. Nanomotors, a new generation of self‐propelled nanocarriers, are capable of delivering and accumulating therapeutic agents within deep inflamed regions through chemical dynamics or photo dynamics [29, 30, 31, 32]. Nanomotor encapsulating therapeutic drugs also safeguard them from rapid biodegradation, resulting in sustained drug release and extended circulation kinetics, and thus spare patients from repeated injection and invasion to disease‐affected areas [33]. Therefore, it is promising to remove the harmful reactivity of ROS by converting the chemical energy stored in the molecules to the movement of nanomotors, leaving only water and oxygen behind [34]. The additional benefit of this strategy is better oxygenation and an increased distribution of the nanomotors in the tissue.

Here, we combined the ROS‐inhibiting effect of metformin with the ROS‐scavenging effect of nanomotors to break the vicious cycle of inflammation that sustains OA at the ROS level (Scheme 1). The nanomotor features a Janus architecture comprising a platinum‐coated gold nanoparticle (AuNP@Pt) “engine” and a periodic mesoporous organosilica (PMO) “container” for MET loading. Leveraging the intrinsic superoxide dismutase (SOD) and catalase (CAT) mimicry of the AuNP@Pt component, the MET‐loaded nanomotors (NM@MET) harness the pathologically elevated ROS within the joint as chemical fuel, enabling autonomous self‐propulsion and deep penetration into the dense matrices of cartilage and synovium. This active transport enables simultaneous “on‐the‐move” ROS scavenging and deep‐tissue MET delivery, which together suppress downstream ROS production in chondrocytes and macrophages. By restoring redox homeostasis, the NM@MET system protects chondrocytes and mitigates synovitis and osteophyte formation, specifically by modulating the interleukin‐induced activation of the NRF2/KEAP1 pathway. Beyond structural preservation, the nanomotors can also alleviate joint pain by suppressing nociceptive signaling in the dorsal root ganglia (DRG). Consequently, this ROS‐fueled, multifunctional nanomotor platform offers a robust strategy for simultaneously arresting cartilage degeneration and resolving OA‐associated pain.

**SCHEME 1:**
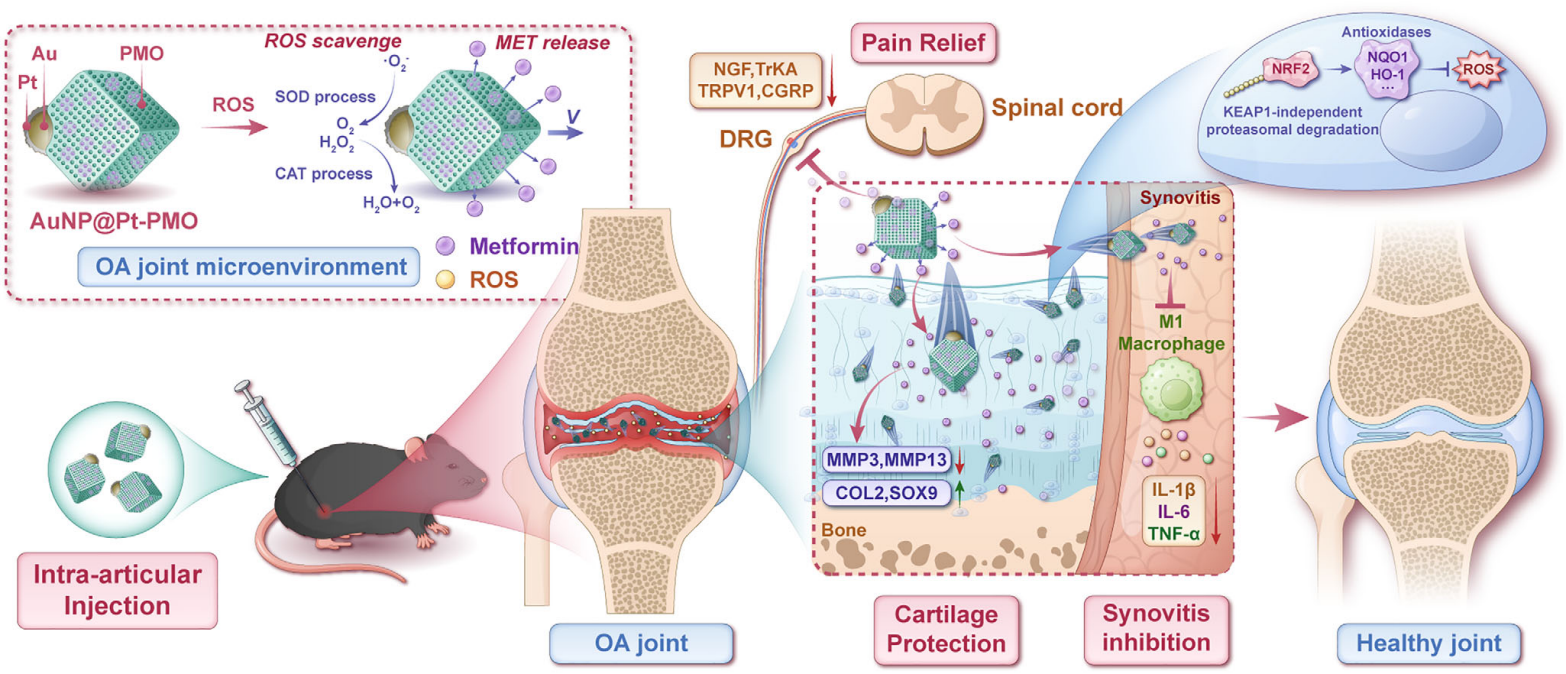
Schematic illustration of the therapeutic mechanism of ROS‐responsive nanomotor loaded with metformin for targeted OA treatment. The Janus‐structured nanomotor harnesses elevated ROS as a chemical fuel to actively penetrate deep into both cartilage and synovial tissue, where it enables sustained MET release. Concurrently, it scavenges excessive ROS and inhibits activation of the NRF2/KEAP1 signaling pathway to protect chondrocytes. Furthermore, NM@MET suppresses nociceptive signaling in DRG, thereby alleviating pain and improving movement in OA mice. Through its multifunctional actions, NM@MET protects articular cartilage, suppresses synovial inflammation, and alleviates joint pain, offering a targeted and responsive strategy for OA treatment.

## 2 Results and Discussion

### 2.1 Synthesis and Characterization of the Nanomotors

As shown in Figure 1A, the AuNP was first prepared, and then Pt nanoseeds were anchored on the surface of Au nanoparticles, forming the core@shell structured AuNP@Pt nanoparticles. Afterward, using the anisotropic island nucleation and growth approach, the PMO nanocubes could nucleate and grow near the AuNP@Pt core@shell nanoparticles to form the AuNP@Pt‐PMO nanocomposites. Subsequently, metformin was absorbed into the PMO nanocube domains' mesopores. The hydrogen peroxide (H_2_O_2_) can be converted to water (H_2_O) and oxygen (O_2_) under the catalysis of Pt, which allows the nanomotors to move by producing a diffusiophoresis force. Transmission electron microscopy (TEM) images displayed the morphology of AuNP and AuNP@Pt (Figure 1B,C). The TEM image in Figure 1D and the scanning electron microscopy (SEM) images in Figure 1E showed that the as‐prepared AuNP@Pt‐PMO nanocomposites, which had a size of around 170 nm, exhibited a clear asymmetric nanostructure. A single‐crystal PMO nanocube (about 120 nm in side length) and a closely related core@shell structured AuNP@Pt nanosphere (approximately 40 nm in diameter) structure the AuNP@Pt‐PMO nanocomposite. Highly organized mesopore channels with a pore width of about 2 nm were present in the single‐crystal PMO nanocube, according to a high‐resolution TEM (HRTEM) picture (Figure 1F). The thickness of the Pt nanoshells on the core@shell AuNP@Pt domain was measured to be ∼10 nm. The successful modification of PMO nanocubes and loading of metformin were confirmed by measuring the zeta potential (Figure 1G). The core design principle of ROS‐driven NM lies in utilizing Janus structures or hollow asymmetric structures, with noble metals or metal oxides serving as catalytic engines [35]. These components convert the overexpressed ROS in the pathological microenvironment into mechanical energy, thereby achieving autonomous motion or enhanced diffusion [36, 37]. To evaluate the drug‐loading capacity of the NM, a standard curve of metformin was constructed based on UV absorption at 233 nm. The loading capacity was determined to be 36 µmol of metformin per mg of NM. The in vitro release profile of metformin from the NM@MET system showed a sustained release behavior over 8 days (Figure S1), confirming the mesoporous structure's drug storage and sustained release capacity. The asymmetric shape and element distribution of a single AuNP@Pt‐PMO nanocomposite were shown by EDS (Figure 1H). It is possible to identify and match the relative locations of all predicted elements, such as Au, Pt, Si, C, and O, in the nanocomposite. ROS are produced at increased levels in osteoarthritis, which can be used as a source for O_2_ production using SOD and CAT‐mimetics [38, 39]. Electron spin resonance (ESR) spectroscopy assay showed that AuNP@Pt‐PMO, either free (NM) or loaded with metformin (NM@MET) could scavenge large amounts of •OH (Figure S2A). Oxygen bubbles produced by NM or NM@MET were visible upon mixing with H_2_O_2_ (Figure S2B). Previous studies have proved the catalytic conversion of superoxide anions (•O_2_^−^) by platinum [40], supporting the SOD/CAT‐like activity of the Pt‐loaded nanozymes.

**FIGURE 1:**
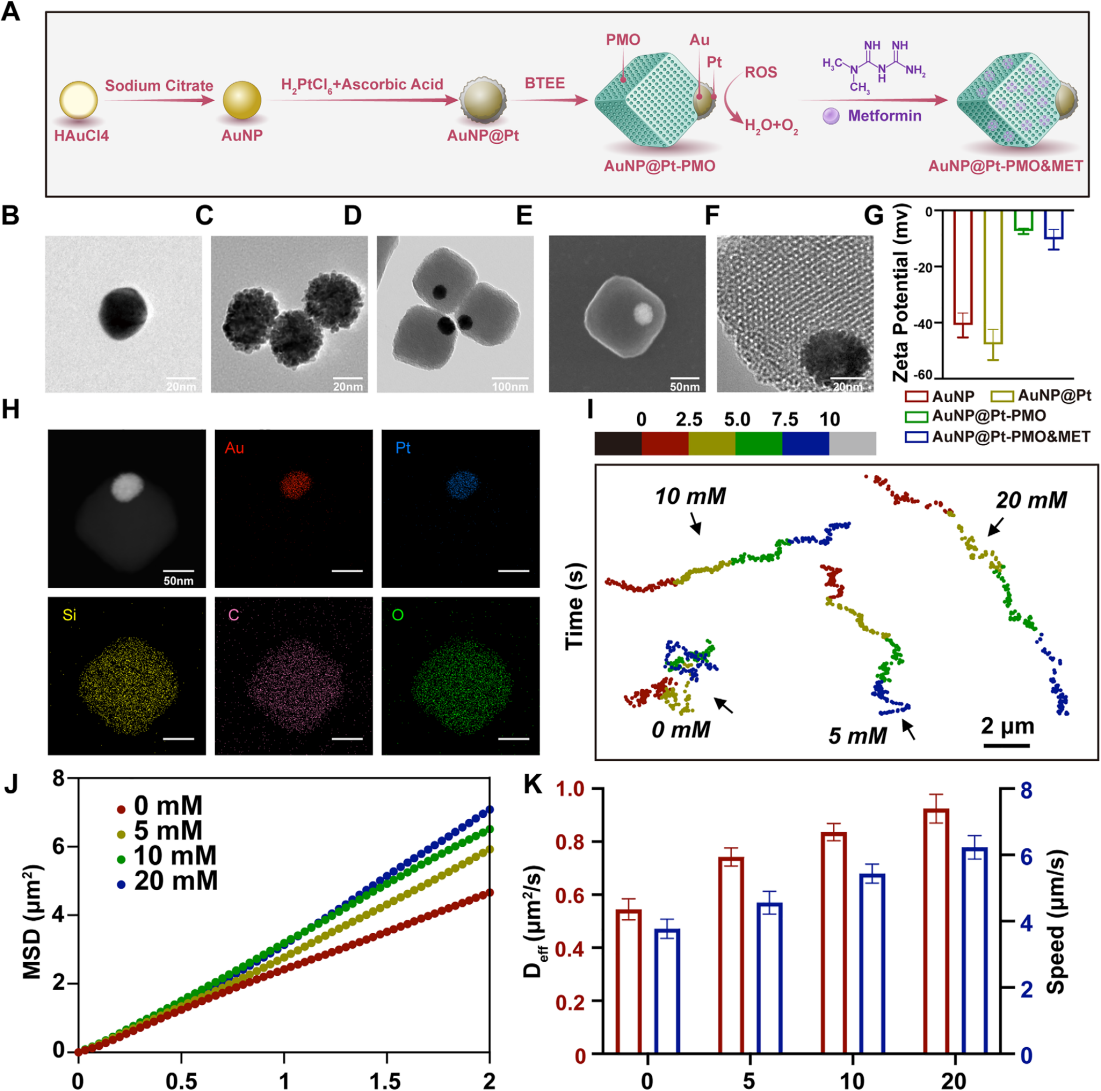
(A) Schematic of the synthesis of NM@MET. TEM images of (B) AuNP, (C) AuNP@Pt, and (D) AuNP@Pt‐PMO. (E) SEM of AuNP@Pt‐PMO. (F) HRTEM images of AuNP@Pt‐PMO. (G) Zeta potentials of various samples. (H) Element mapping of AuNP@Pt‐PMO nanomotors. (I) Time‐lapse trajectories (in 10 s), (J) MSD, and (K) D_eff_ and corresponding mean speeds of the nanomotors in H_2_O_2_ solution at varying concentrations.

The self‐propelled movement of the nanomotors in H_2_O_2_ is examined by trajectory analysis. The action of the nanomotors is directed and can be augmented by raising the concentration of H_2_O_2_ (Figure 1I). At different fuel concentrations, the Janus nanomotors' mean square displacement (MSD) is calculated as a function of time interval (Δt). The MSD exhibits a linear rise with Δt and biofuel concentration (Figure 1J), signifying motility that is reliant on biofuel concentration. When the H_2_O_2_ concentration rises from 0 to 5, 10, and 20 mm, respectively, the corresponding effective diffusion coefficient (D_eff_) rises from 0.53 to 0.75, 0.83, and 0.92 µm^2^ s^−1^ (Figure 1K). By adjusting the biofuel content from 0 to 20 mm, the average speed of the nanomotors (v = s/t, where s is the travel distance in time t) may be controlled between 3.85 and 6.25 µm/s (22.65–36.76 body‐length/s) (Figure 1K). The outcomes show that the biofuel H_2_O_2_ can power the precisely crafted enzyme‐based nanomotors, and that altering the biofuel concentration can successfully regulate the motion of the nanomotors. The propulsion characteristics of Janus nanomotors in a physiological environment (DMEM medium) were also examined for mimicking biomedical applications (Figure S3). The trajectory analysis indicated that the activity of nanomotors is directional and may be enhanced by increasing the concentration of H_2_O_2_ in DMEM medium (Figure S3A). The MSD results indicate that the motility of nanomotors remains dependent on biofuel concentration (Figure S3B). As the concentration of H_2_O_2_ increases from 0 to 5, 10, and 20 mm, the effective diffusion coefficient (D_eff_) increases from 0.37 to 0.58, 0.66, and 0.76 µm^2^ s^−^^1^, respectively. Modifying the biofuel concentration from 0 to 20 mm allows for the regulation of nanomotor velocity between 3.56 and 5.69 µm/s (20.94–33.47 body‐lengths per second) (Figure S3C). The results indicated that the self‐propulsion of the Janus nanomotors was merely slightly influenced in the physiological environment.

### 2.2 NM Shows Enhanced Cartilage Penetration and Intra‐Articular Retention in OA Joints

To assess the in vivo motion behavior of NMs, FITC‐labeled NMs were injected into the knee joint of a rat subjected to DMM surgery. It's evident that in both the Sham and DMM groups, the NMs enabled deeper penetration of the fluorescent probe into the cartilage compared with free FITC, with a more prominent penetration observed in the DMM group (Figure 2A,B). These results confirmed that the NM synthesized in this study can successfully carry the drug to the deep layer of cartilage tissue in the OA microenvironment. The retention profile of NM in knee joints was further assessed by ICP‐MS detection of Au, Pt, and Si. The relative levels of these elements gradually decreased over 21 days after intra‐articular injection, indicating progressive clearance of NM from the joint. Notably, DMM joints exhibited higher Au, Pt, and Si retention than Sham joints during the early stage, suggesting enhanced retention of NM in the OA joint microenvironment (Figure 2C). In vivo IVIS imaging further confirmed the prolonged joint retention of NM. After intra‐articular injection, free Cy5.5 was rapidly cleared from the knee region and showed earlier fluorescence accumulation in the bladder area, indicating fast elimination. In contrast, Cy5.5‐NM displayed a more sustained fluorescence signal at the injected knee joint. The signal of Cy5.5‐NM remained detectable for approximately 7 days in Sham joints and was further prolonged to 14 days in DMM joints (Figure 2D). This extended retention in DMM mice suggests that the pathological OA microenvironment, characterized by disrupted cartilage matrix and increased tissue permeability, facilitates NM penetration and residence within the joint. Consistent with the ICP‐MS results, the fluorescence intensity of Cy5.5‐NM gradually decreased over time, indicating progressive clearance of NM‐associated components from the joint cavity.

**FIGURE 2:**
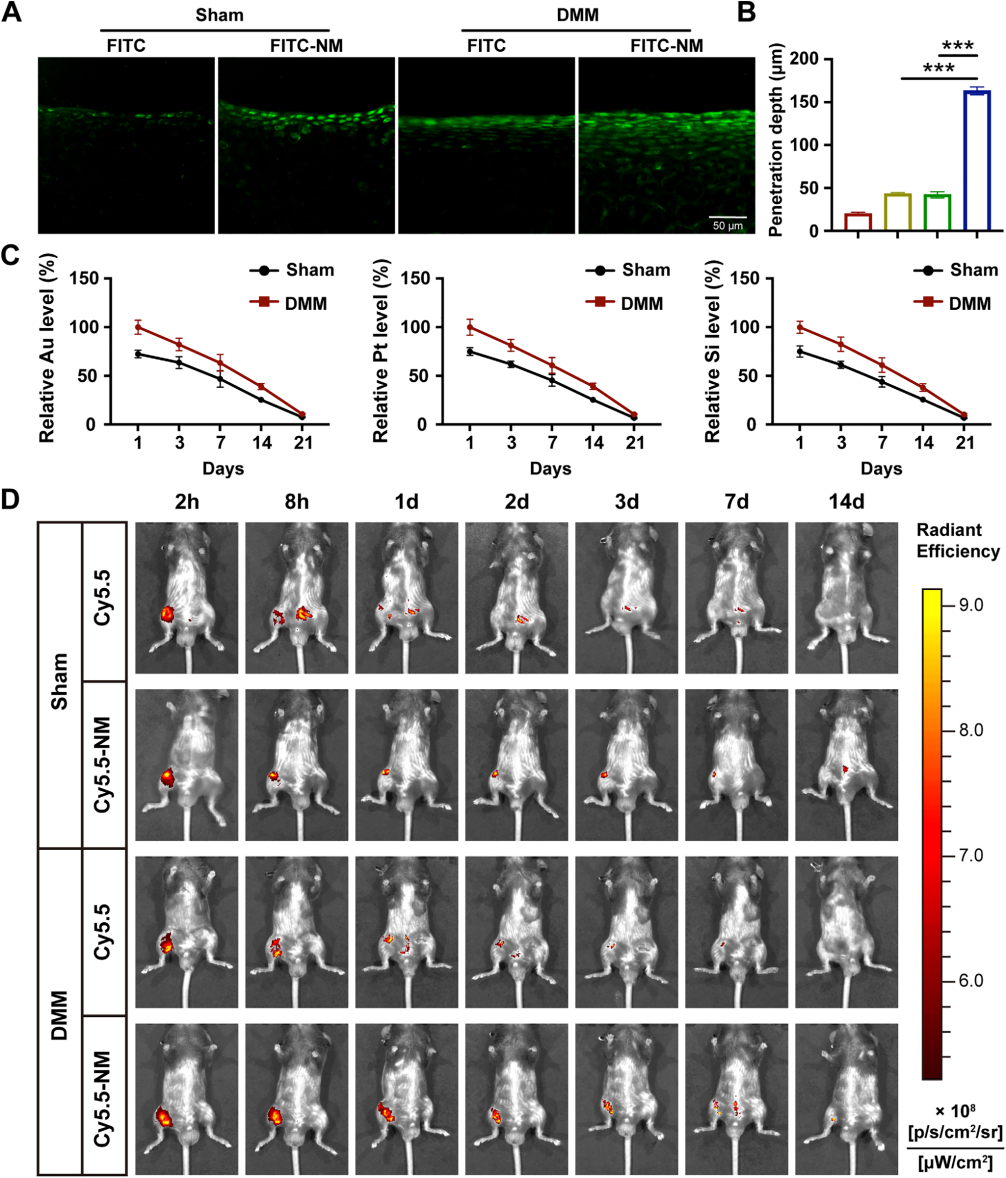
(A) Representative fluorescence images of FITC‐labeled NMs spread in rat cartilage tissues and (B) quantification of the penetration depths. (C) Time‐dependent retention of Au, Pt, and Si in mouse knee joints after intra‐articular injection of NM, as determined by ICP‐MS. (D) Representative in vivo IVIS fluorescence images of Sham and DMM mice after intra‐articular injection of free Cy5.5 or Cy5.5‐labeled NM. Data are shown as mean ± SEM. ^***^ is for *p* < 0.001.

### 2.3 Cytotoxicity, ROS Scavenging, and Chondroprotection of NM@MET

In vitro experiments were performed on primary murine chondrocytes and the macrophage cell lineRAW264.7 cells to assess the biological effects of NM@MET (Figure 3A). We next investigated the cytotoxicity of NM@MET to chondrocytes through the CCK‐8 cell viability assay. The viability of chondrocytes maintained at 90% or above after incubation with the NM@MET for 1, 3, 5d at a concentration as high as 1 mg ml^−1^, suggesting the low cytotoxicity of the NM@MET (Figure 3B). Live/dead staining showed that NM@MET could effectively inhibit cell apoptosis (Figure 3C,D). NM@MET demonstrated comparable biosafety in RAW264.7 cells (Figure S4). Evidence suggests a disparity between ROS production and the antioxidant capacity of chondrocytes, contributing to cartilage deterioration and chondrocyte apoptosis [41]. To assess the ROS scavenging and anti‐inflammatory properties of the NM@MET nanomotors, we employed H_2_O_2_ (100 µm)‐treated chondrocytes to replicate ROS‐damaged cartilage in vitro [42]. The results indicated a modest red fluorescence in the control group (Figure 3E,F), which was markedly amplified upon induction by H_2_O_2_, signifying the generation of substantial quantities of ROS by H_2_O_2_. The fluorescence intensity of the MET‐treated group exhibited a slight reduction relative to the H_2_O_2_ group, indicating a limited scavenging action on intracellular ROS. ROS are also crucial in macrophage‐mediated acute inflammation and function as signaling molecules that modulate M1 macrophage polarization [43]. M1 macrophage‐induced synovitis exacerbates the degenerative progression of osteoarthritis [44]. ROS levels were markedly elevated in RAW264.7 cells induced to M1 polarization with LPS (100 ng ml^−1^) and IFN‐γ (20 ng ml^−1^) treatment, and both NM and NM@MET effectively decreased ROS generation, with NM@MET demonstrating superior efficacy (Figure S5). To clarify whether nanomotors can effectively reduce the inflammatory response of chondrocytes, we investigated gene expression using the qPCR method (Figure 3G–J). After interleukin‐1β (IL‐1β) stimulation, mouse chondrocytes were incubated with different concentrations of NM@MET from 31.25 to 250 µg ml^−1^. IL‐1β stimulation markedly increased the mRNA levels of *Mmp3* and *Mmp13* while decreasing *Col2a1* and *Sox9* levels. NM@MET dose‐dependently reversed the expression of these markers, with the lowest expression at 125 µg ml^−1^ and no longer decreased at 250 µg ml^−1^. Comparable tests were conducted with RAW264.7 cells, revealing that NM@MET inhibited the release of inflammatory cytokines, such as IL‐1β and IL‐6, from M1 macrophages in a concentration‐dependent manner (Figure S6). Western blot was performed to investigate the sustained release effect of nanomotors (Figure 3K–O). Chondrocytes were incubated with NM@MET or MET for 1, 3, 5 d in an inflammatory environment. The anti‐inflammatory effect of the MET group diminished over time. However, NM@MET exhibited stable, protective effects that lasted for five days. Similar experiments were conducted in RAW264.7 cells. The inhibitory effect of NM@MET on the inflammatory phenotypes of M1 macrophages was more prolonged and stable than that of MET, indicating that nanomotors serve as an effective drug delivery system (Figure S7).

**FIGURE 3:**
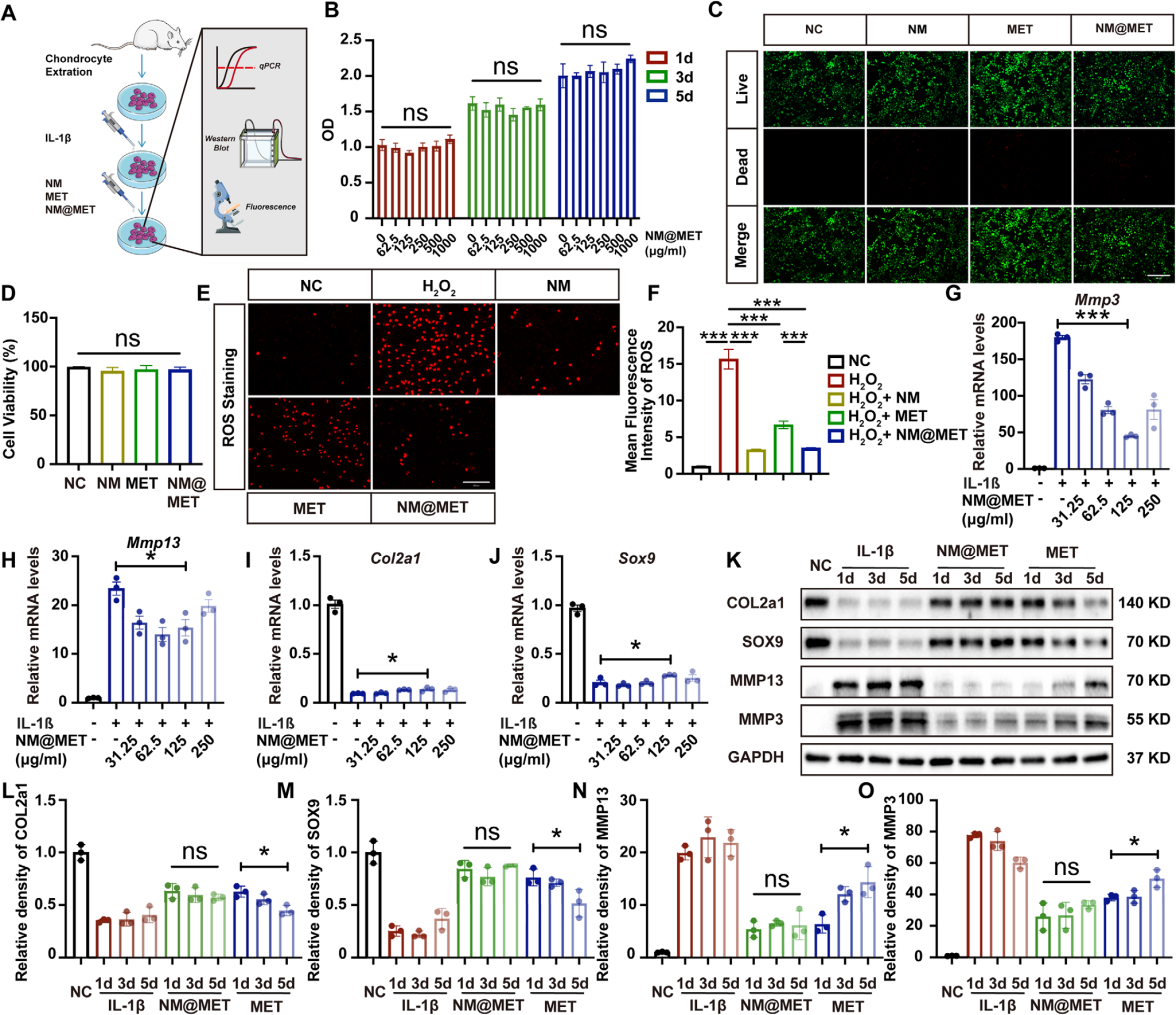
(A) Illustration of in vitro experiments on mouse chondrocytes. (B) Cytotoxicity of NM@MET after 1, 3, 5d co‐incubation with chondrocytes. (C) Calcein‐AM/PI staining images and (D) quantification of dead and living cells treated with NM, MET, and NM@MET. (E) Fluorescence images and (F) intensity of DHE. (G–J) qPCR determined mRNA levels. (K) Representative western blot and (L–O) quantification analysis of MMP3, MMP13, COL2a1, and SOX9. n = 3 independent experiments. Data are shown as mean ± SEM. ^*^ is for *p* < 0.05, ^**^ for *p* < 0.01, ^***^ for *p* < 0.001, and ns > 0.05 respectively.

### 2.4 NM@MET Regulates Neuroactive Pathway and NRF2/KEAP1 Pathway

To investigate the molecular mechanism of NM in protecting chondrocytes, we performed RNA sequencing of IL‐1β‐treated chondrocytes (IL group), and IL‐1β‐induced chondrocytes treated with NM and NM@MET (Figure 4A). Differentially expressed genes (DEGs) were identified using an adjusted p‐value of less than 0.05. Genes with absolute logarithmic fold change (log2FC) values greater than 1.0 were included in the final DEGs. The results showed that 189 genes were upregulated and 201 genes were downregulated in the NM group compared to the IL group (Figure 4B). The NM@MET vs IL group exhibited 187 upregulated genes and 804 downregulated genes (Figure S8), while the comparison between NM@MET and NM resulted in 149 upregulated and 640 downregulated genes (Figure 4C). The heatmap indicated a reduction in inflammatory markers such as *Mmp3*, *Mmp13*, *Nos2*, and *Ptgs2* in the NM group, with a more pronounced decrease observed in the NM@MET group (Figure 4D). According to GO and KEGG enrichment analysis results, the NM intervention induced DEGs in chondrocytes were significantly enriched in postsynaptic specialization, NADPH oxidase complex, and neuroactive ligand‐receptor interaction (Figure 4E,F). The GO and KEGG analyses comparing NM@MET with NM alone revealed that MET treatment also enriched multiple neuro‐related pathways, indicating a combined effect of NM and MET in neural regulation (Figure 4G,H). The gene set enrichment analysis (GSEA) for NM vs IL showed that DEGs were tightly associated with glutathione metabolism and neuroactive ligand‐receptor interaction (Figure 4I,O). Heatmap showed glutathione metabolism‐related markers declined in the NM and NM@MET groups (Figure 4J). Several antioxidant systems regulating levels of ROS depend on glutathione [45]. The result indicated that NM and NM@MET are closely related to ROS. To further substantiate the RNA‐sequencing results, a western blot was conducted to test the protein expression of NRF2, KEAP1, and HO1 in chondrocytes in the presence of NM or NM@MET with or without IL‐1β (Figure 4K). NRF2 is a transcription factor that binds to antioxidant response elements to activate the expression of antioxidant genes such as HO1 [46]. KEAP1 degrades NRF2 through the ubiquitination pathways. The NRF2/KEAP1 pathway is a crucial antioxidant defense system that shields cells from oxidative damage [47]. NM and NM@MET diminished the expression of NRF2 and HO1 that was upregulated by IL‐1β and improved the expression of KEAP1. These findings demonstrated that nanomotors diminish ROS production and restore the oxidative balance, thereby modulating NRF2/KEAP1 pathway activation. To verify whether NRF2 signaling contributes to NM@MET‐mediated chondroprotection, IL‐1β‐stimulated chondrocytes were treated with NM@MET in the presence or absence of the NRF2 inhibitor ML385. NM@MET restored COL2a1 expression and reduced MMP13 expression. ML385 did not obviously affect NRF2 protein expression but decreased the expression of the downstream antioxidant protein HO1, indicating inhibition of NRF2‐mediated antioxidant activity. Moreover, ML385 partially reversed the protective effects of NM@MET, as shown by reduced COL2a1 expression, increased MMP13 expression, and elevated ROS accumulation compared with NM@MET treatment alone (Figure 4L–N). These findings indicate that NRF2 downstream antioxidant signaling is involved in the ROS‐scavenging and chondroprotective effects of NM@MET.

**FIGURE 4:**
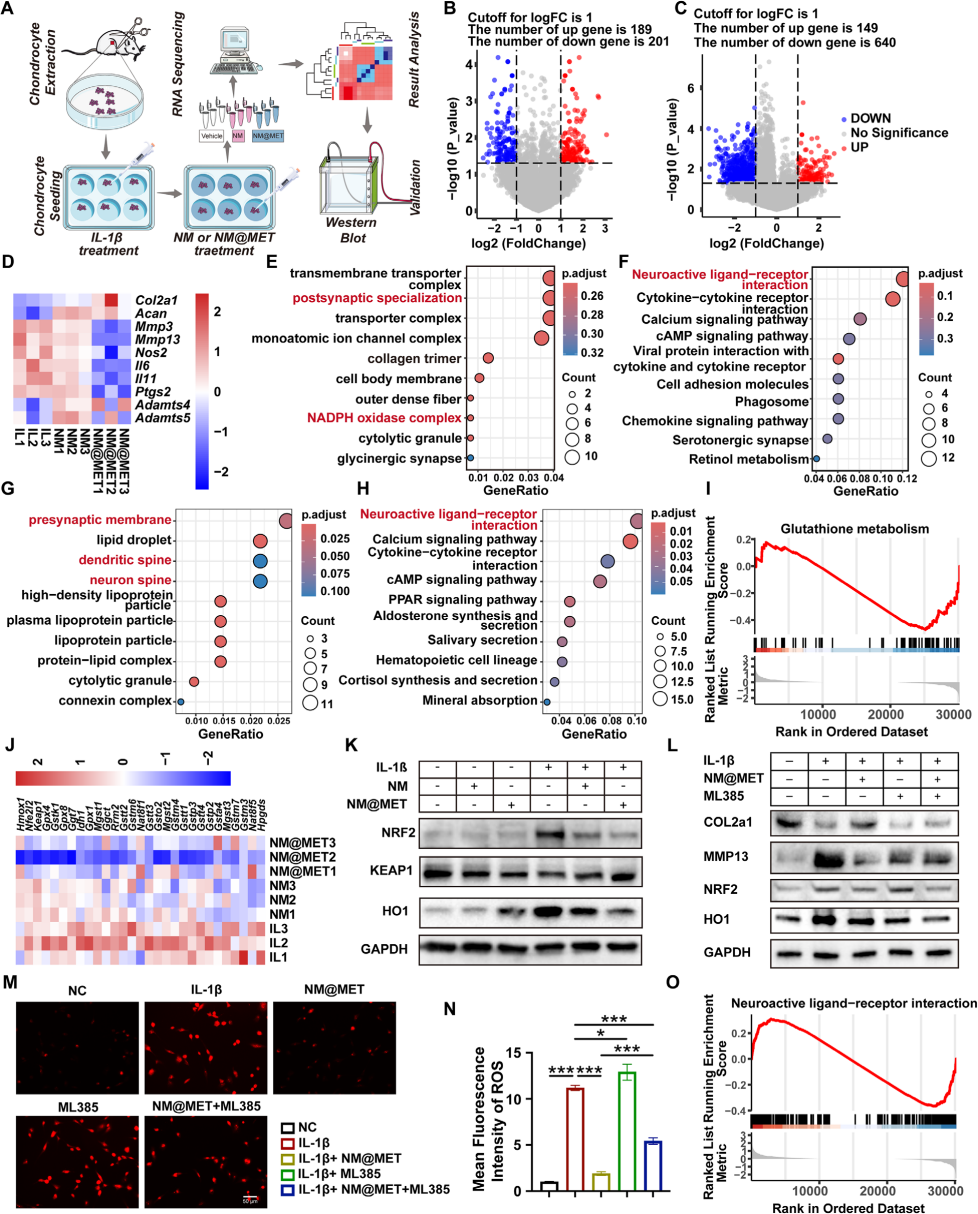
(A) Schematic of the process of RNA‐Seq. (B) Volcano plot of DEGs of the NM group vs IL group. (C) Volcano plot of DEGs of NM@MET group vs NM group. (D) The heat map showed inflammation‐associated markers. (E) GO terms analysis result of the DEGs of the NM group vs the IL group. (F) KEGG enrichment analysis result of the DEGs of the NM group vs the IL group. (G) GO terms analysis result of the DEGs of the NM@MET group vs the NM group. (H) KEGG enrichment analysis result of the DEGs of the NM@MET group vs the NM group. (I) GSEA was used to identify the distribution of genes in the glutathione metabolism pathway. (J) Heat map of DEGs that were enriched in glutathione metabolism. (K) Western blot result revealed the protein expression of NRF2, KEAP1, and HO1. (L) Western blot analysis of COL2a1, MMP13, NRF2, and HO1 expression in chondrocytes treated with IL‐1β, NM@MET, and/or the NRF2 inhibitor ML385. (M) Representative fluorescence images and (N) quantification of intracellular ROS levels in chondrocytes after different treatments. (O) GSEA was used to identify the distribution of genes in the neuroactive ligand‐receptor interaction pathway.

ROS are highly reactive chemicals that can substantially exacerbate joint pain by inducing inflammation and damaging cartilage and bone tissue inside the joint due to their oxidative stress effects when created in excess [48]. The enrichment analysis of sequencing data revealed that NM and NM@MET treatments were significantly associated with postsynaptic specialization and neuroactive ligand‐receptor interaction pathways. Therefore, we predicted that NM and NM@MET may modulate neuronal signaling to mitigate arthritic pain.

### 2.5 In Vivo NM@MET‐Mediated OA Therapy

Cartilage tissue in joints, primarily hyaline cartilage, containing densely packed collagen fibers aligned in different patterns at different layers, allows smooth, low‐friction movement between bones and to absorb shock, reducing wear and tear during motion [49]. The chondrocyte and the extracellular matrix formed a thick and dense tissue layer to limit the substance exchange between the subchondral bone and the joint cavity, which also maintains the joint cavity as a hypoxic environment [50]. The IA injection, as a typical local treatment technique, normally allows the drugs to affect the tissue inside the joint cavity. However, these drugs can only function on the superficial layer of the joint cartilage. It's important to note that OA does not solely affect the surface layer of cartilage, but is a degenerative joint disease that involves the gradual breakdown of the entire articular cartilage and changes in the subchondral bone environment, leading to changes in its composition, structure, and function [51]. Thus, nanocarriers show great potential in the field of drug delivery, so as to increase the penetration depth of cartilage tissue. During the pathological process, the ECM of cartilage becomes loose and shows increased permeability [52]. The ROS in the microenvironment provides as the fuel for nanomotor movement.

Given that the NM@MET exhibits favorable antioxidant and anti‐inflammatory activities in vitro, we assessed its treatment efficacy in vivo using a DMM‐induced OA mouse model. NM@MET was delivered into the knees of DMM mice every 7 days by intra‐articular injection. For comparison, healthy mice underwent sham surgery (negative control), and OA mice were injected with PBS (positive control), NM, MET, or NM@MET (Figure 5A). The onset of inflammation is usually accompanied by an increase in knee joint temperature due to the induced synovitis, therefore the thermographic effects of the knee joints were assessed using an FLIR infrared thermal camera after different treatments [53]. The temperature of knee joints in DMM mice in the PBS (positive control) group reached as high as 36.3°C, while this increase was attenuated in different degrees after the OA mice were treated with NM, MET, or NM@MET. As displayed in Figure 5B,C, the temperature in the NM@MET group was the lowest (35.4°C, p < 0.05, among all groups except the PBS negative control group), implying the best anti‐inflammatory efficacy of the NM@MET. To investigate the underlying mechanism, how the inflammation was reduced, the luminescence probe L‐012 was injected into the mice, and an in vivo imaging system was utilized to assess the distribution of ROS. As shown in Figure 5D,E, the luminescence of inflamed knee joints in the PBS‐positive control group is much stronger than that of healthy mice in the negative PBS control group. Already, NM alone significantly eliminated ROS in vivo. The luminescence intensity in the NM@MET group was the weakest, suggesting an independent, additive effect of NM and MET on ROS‐reduction. Bone remodeling, characterized by osteophyte production and subchondral bone sclerosis, is one of the primary pathological hallmarks of osteoarthritis, which follows the pathophysiology of articular cartilage deterioration [54]. To assess the comprehensive protective effect of NM@MET on the knee joint, we conducted a further analysis of the bone condition in the knee joint of mice. Micro‐computed tomography (Micro‐CT) of the mouse knee joint revealed an elevated quantity of osteophytes and subchondral bone sclerosis following DMM (Figure 5F,G). Mice treated with NM@MET showed improvement in these pathological alterations. Subchondral bone sclerosis was indicated by micro‐CT analysis of the subchondral bone region, which revealed that DMM mice had higher bone volume/total volume (BV/TV) (Figure 5H), trabecular number (Tb.N) (Figure 5I), and decreased trabecular separation (Tb.Sp) than mice in the sham group (Figure 5J). Trabecular thickness exhibited no significant variation among the groups (Figure 5K). NM@MET therapy resolved the subchondral abnormalities linked to OA, suggesting that these bone alterations are secondary to the inflammation of OA.

**FIGURE 5:**
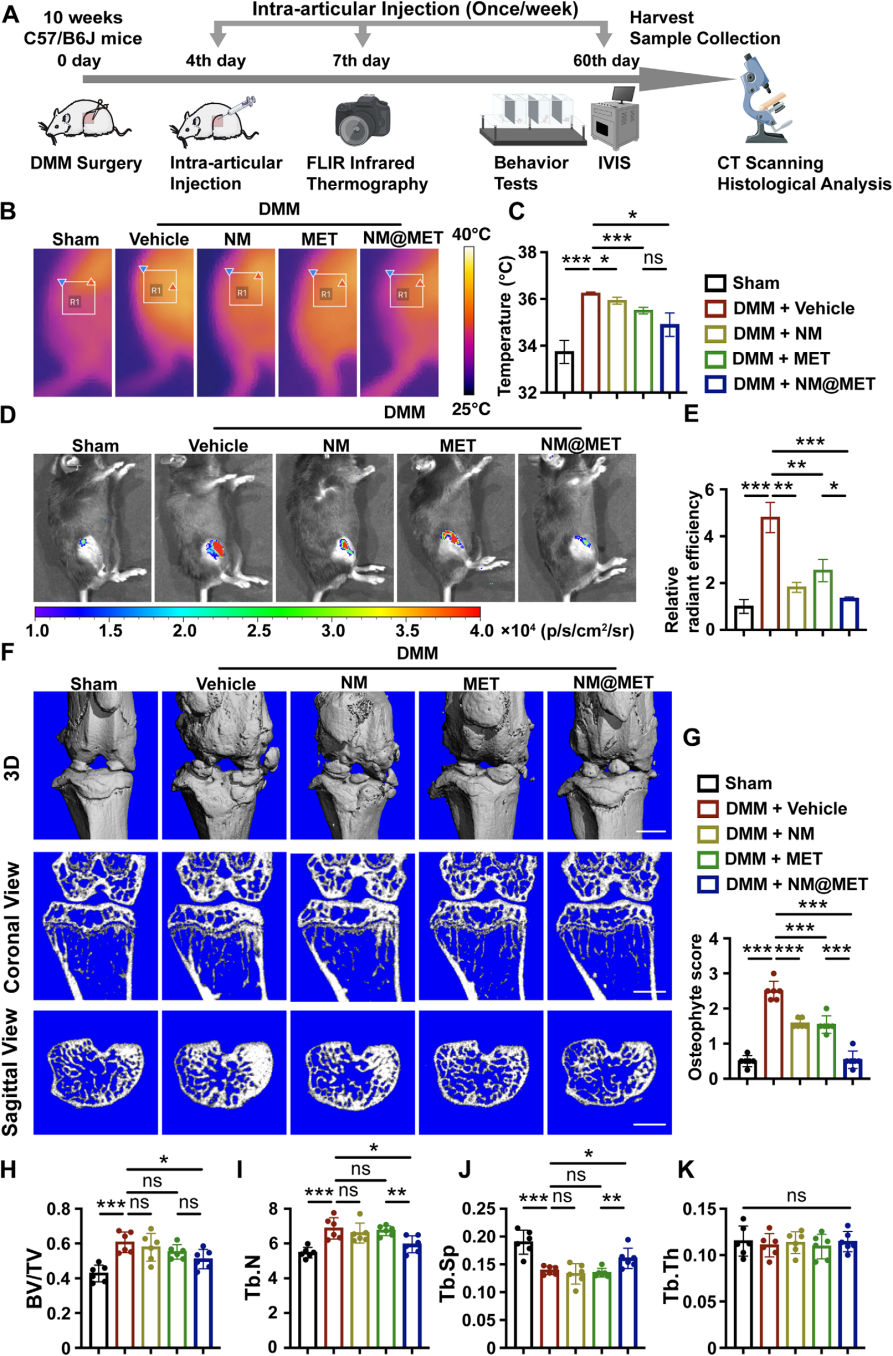
(A) Treatment schedule of an OA mouse model in vivo. (B) Representative thermographic scans of healthy knees and DMM knees subjected to various therapies. (C) Quantification of temperature in healthy knees or OA knees after treatments with different formulations (n = 3). (D) In vivo luminescence images and (E) the relative L‐012 luminescence intensity of healthy knees and OA knees in mice after different treatments (n = 3). (F) Representative micro‐CT reconstructions and sectional views (coronal and sagittal) of knee joints in normal mice or OA mice after treatment. Scale bar: 1.0 mm. Quantitative analyses of (G) osteophyte scoring, (H) BV/TV, (I) Tb. N, (J) Tb.Sp, and (K) Tb. Th. of subchondral bone in different groups (n = 6). Data are shown as mean ± SEM. ^*^ is for p < 0.05, ^**^ for p < 0.01, ^***^ for p < 0.001, and ns > 0.05 respectively.

Beyond therapeutic efficacy, systemic biosafety was assessed by serum biochemical analysis in healthy mice and histological examination of major organs in Sham and DMM mice. In healthy mice, intra‐articular administration of Vehicle, NM, MET, or NM@MET did not significantly alter serum alanine aminotransferase (ALT), aspartate aminotransferase (AST), total bilirubin (TBIL), urea, or creatinine (CREA) levels, indicating no detectable hepatic or renal dysfunction (Figure S9A). Consistently, H&E staining of the heart, liver, spleen, lung, and kidney showed no obvious histopathological abnormalities in Sham mice or DMM mice receiving different treatments, including NM@MET (Figure S9B). These results suggest that NM@MET exhibits favorable systemic biosafety in vivo.

### 2.6 NM@MET Attenuates Cartilage Degradation and Synovitis

The destruction of articular cartilage is mainly ascribed to the gradual loss of ECM. We validated the function of NM@MET in safeguarding articular cartilage by evaluating its integrity. The Safranin‐O/Fast green (S.O.) staining demonstrated significant cartilage deterioration and a marked decrease in cartilage thickness following DMM surgery (Figure 6A). Remarkably, following NM@MET therapy, the deterioration of articular cartilage was mitigated, as evidenced by a reduced Osteoarthritis Research Society International (OARSI) score (Figure 6B), an elevated total chondrocyte count, and enhanced total cartilage thickness. Moreover, in the MET and NM@MET treatment groups, an increase in COL2a1+ area and a decrease in MMP3+ and MMP13+ chondrocyte percentages were seen (Figure 6C–H), indicating their capacity to enhance cartilage anabolism and reduce cartilage catabolism. To further address the heterogeneity of human OA and validate the therapeutic relevance of NM@MET beyond the DMM mouse model, human normal cartilage and OA cartilage explants were cultured ex vivo and treated with NM, MET, or NM@MET. In normal human cartilage explants, Safranin O/Fast Green and immunohistochemical staining showed that NM@MET did not cause obvious proteoglycan loss, matrix disruption, or abnormal increase in catabolic marker expression, indicating good compatibility with normal cartilage (Figure S10A, B). In human OA cartilage explants, NM@MET markedly enhanced proteoglycan retention and COL2a1 expression while reducing ADAMTS5 and MMP13 expression compared with Vehicle treatment (Figure S10C, D). Collectively, our findings demonstrate that NM@MET had a more potent chondroprotective effect than MET in mice with DMM‐induced osteoarthritis and human OA cartilage tissue. Hematoxylin‐eosin (H&E) staining was also used to assess inflammatory cell infiltration in the joints' synovial and adipose tissue (Figure 6I). Healthy mice had a normal synovial interstitial cell structure with a single layer of lining cells. In contrast, the number of lining cells and stromal cells on the synovial surface of OA mice significantly increased, indicating obvious synovitis in the joints. There was almost no inflammatory cell infiltration in the synovial tissue in the NM@MET‐treated knee joints, confirming the strong anti‐inflammatory effect of NM@MET on synovitis (Figure 6J). We further discovered an accumulation of macrophages in osteoarthritis synovial tissue. Following DMM surgery, there was an increase in F4/80 and iNOS‐positive macrophages (inflammatory M1 type), whereas CD206‐positive macrophages (anti‐inflammatory M2 type) remained constant. In the NM@MET treatment group, the proporsion of M1‐specific iNOS+ macrophages drastically diminished, while the level of M2‐specific CD206+ macrophages remained constant in comparison to the control group. The nanomotor marginally reduced cartilage degeneration and diminished infiltration of M1 macrophages. Metformin had a more pronounced effect than the nanomotor, albeit not as potent as NM@MET, again suggesting an independent additive effect (Figure 6K–P).

**FIGURE 6:**
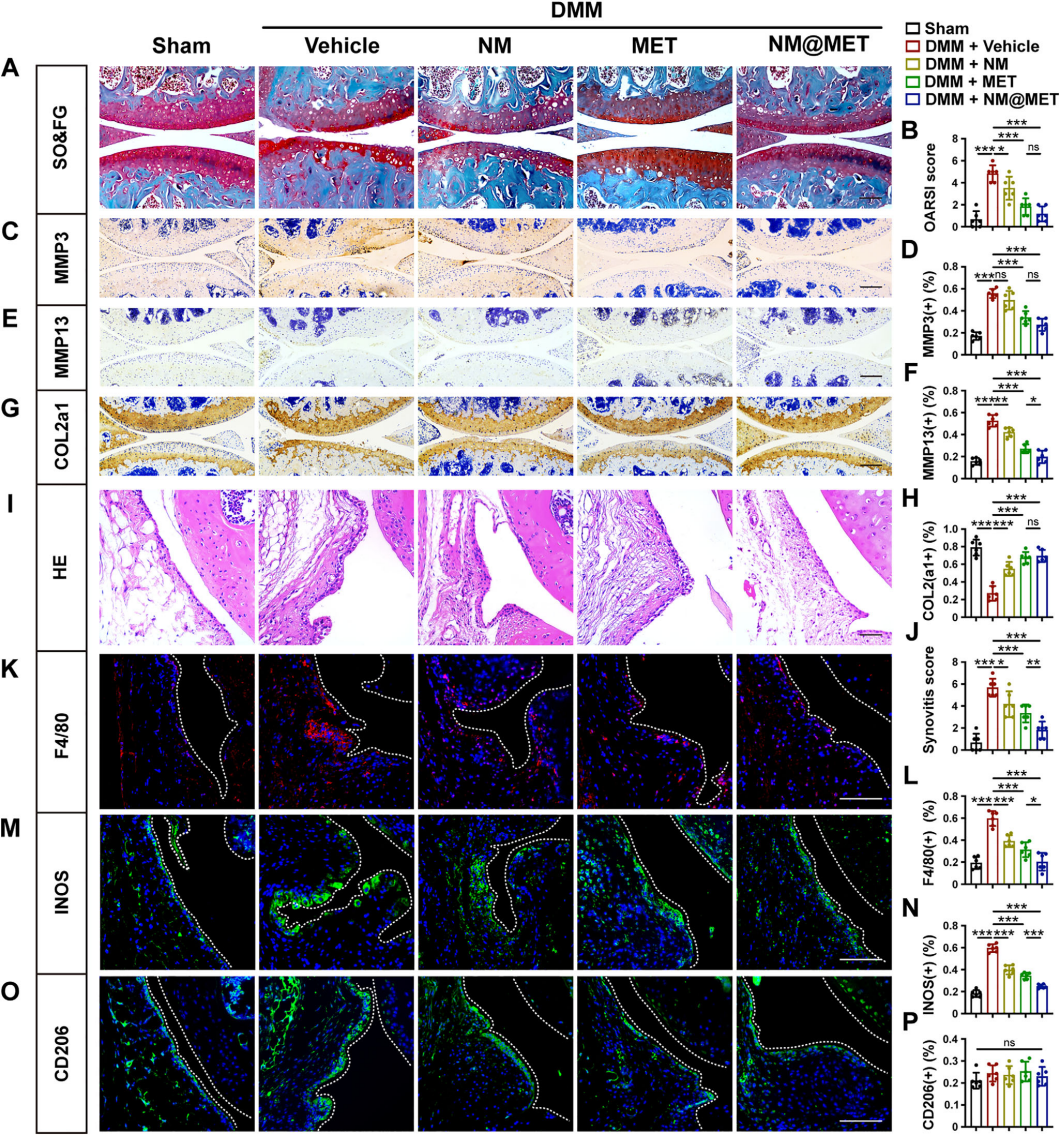
(A) Safranin O/Fast Green staining of knee joints of each group, and (B) OARSI scores. Scale bar: 100 µm. Representative images of immunohistochemistry of (C) MMP3, (E) MMP13, and (G) COL2a1. Scale bar: 200 µm. (D, F, H) Quantification of MMP3, MMP13, and COL2a1‐positive cells in cartilage. (I) H&E staining of synovial tissues and (J) synovitis scoring. Scale bar: 50 µm. Representative images of immunofluorescence of (K) F4/80, (M) iNOS, and O) CD206. Scale bar: 100 µm. (L, N, P) Quantification of F4/80, ikNOS, and CD206‐positive cells in synovium. n = 6. Data are shown as mean ± SEM. ^*^ is for *p* < 0.05, ^**^ for *p* < 0.01, ^***^ for *p* < 0.001, and ns > 0.05 respectively.

### 2.7 NM@MET Inhibits DRG‐Related Peripheral Pain Sensitivity and Improves Behavior Performance in Mice

The peripheral nociceptive system plays an important part in OA‐pain perception. NGF/TrkA signaling is a primary route that regulates the hierarchical development and postnatal synaptic plasticity of nociceptive neurons [55]. These chemicals collaboratively contribute to the survival, differentiation, and adaptability of the peripheral nervous system. In accordance with this concept, DRG sensory neurons in mice suffering from OA pain have heightened immunofluorescence signals for NGF/TrkA‐dependent pain‐related downstream pathways associated with mechanical allodynia and heat nociception [56]. In osteoarthritis mice, heightened levels of nerve growth factor (NGF) and tropomyosin receptor kinase A (TrkA) in DRG sensory neurons correspond with an increase in the transient receptor potential vanilloid 1 (TRPV1) protein and calcitonin gene‐related peptide (CGRP). Given that chondrocyte transcriptomic analysis revealed enrichment of neuroactive ligand‐receptor interaction pathways, and previous studies have supported the use of chondrocyte–neural cell interaction models to investigate neuro‐cartilage communication in OA pain [57], we next investigated whether inflammatory chondrocytes could directly induce neuronal activation. Chondrocytes were stimulated with IL‐1β and treated with NM, MET, or NM@MET, and the collected conditioned medium was used to stimulate ND7/23 sensory neuron‐like cells. Immunofluorescence staining showed that conditioned medium from IL‐1β‐stimulated chondrocytes markedly increased FOS, NGF, TrkA, CGRP, and TRPV1 expression in ND7/23 cells, indicating enhanced neuronal activation and nociceptive signaling (Figure S11A, B). In contrast, conditioned medium from NM@MET‐treated chondrocytes significantly reduced the expression of these markers, showing the strongest overall inhibitory effect among the treatment groups. These results suggest that NM@MET alleviates the pro‐nociceptive effect of inflammatory chondrocytes on sensory neuron‐like cells, thereby supporting a direct chondrocyte–neuronal communication mechanism involved in OA pain modulation. We hypothesized that a positive effect of NM@MET on pain would therefore mitigate these alterations. Indeed, we found markedly reduced levels of NGF, TrkA, and the downstream targets CGRP and TRPV1 in DRG tissues (Figure 7A–H), suggesting suppression of the nociceptive system.

**FIGURE 7:**
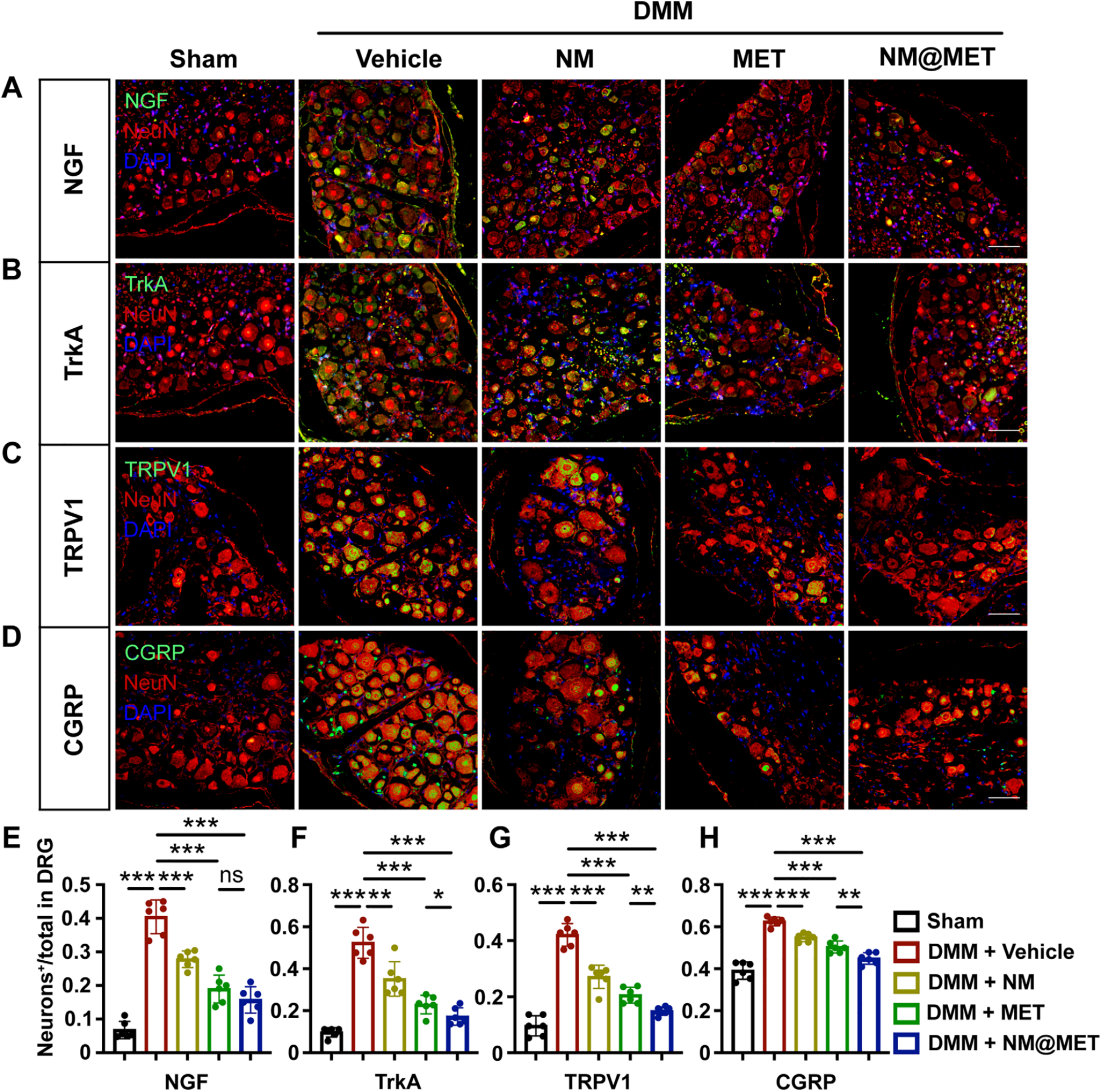
Histological slices of the innervating lumbar DRG (L3/L5) in mice were subjected to immunofluorescence analysis 60 days after DMM. The nuclear protein NeuN/RBFOX3, which is unique to neurons, was used to label DRG sensory neurons. (A–D) Representative images of immunofluorescence of NGF, TrkA, TRPV1, and CGRP. Scale bar: 50 µm. (E–H) Quantification of NGF, TrkA, TRPV1, and CGRP positive cells in DRG. n = 6. Data are shown as mean ± SEM. ^*^ for *p* < 0.05, ^**^ for *p* < 0.01, ^***^ for *p* < 0.001, and ns > 0.05 respectively.

To test if there is a clinical correlate to our findings in nociceptive nerves, we conducted an analysis of the mice's physical activities using behavioral studies to assess knee function. The von Frey fiber test results indicated a significant reduction in paw withdrawal response thresholds in mice post‐DMM surgery. The treatments reversed reduced paw withdrawal response thresholds (Figure 8A). Already, NM treated mice exhibited a slight improvement compared to DMM animals, and the efficacy of MET was marginally superior to that of NM. Again, NM@MET had the strongest effect, with withdrawal response thresholds similar to those of untreated control animals.

**FIGURE 8:**
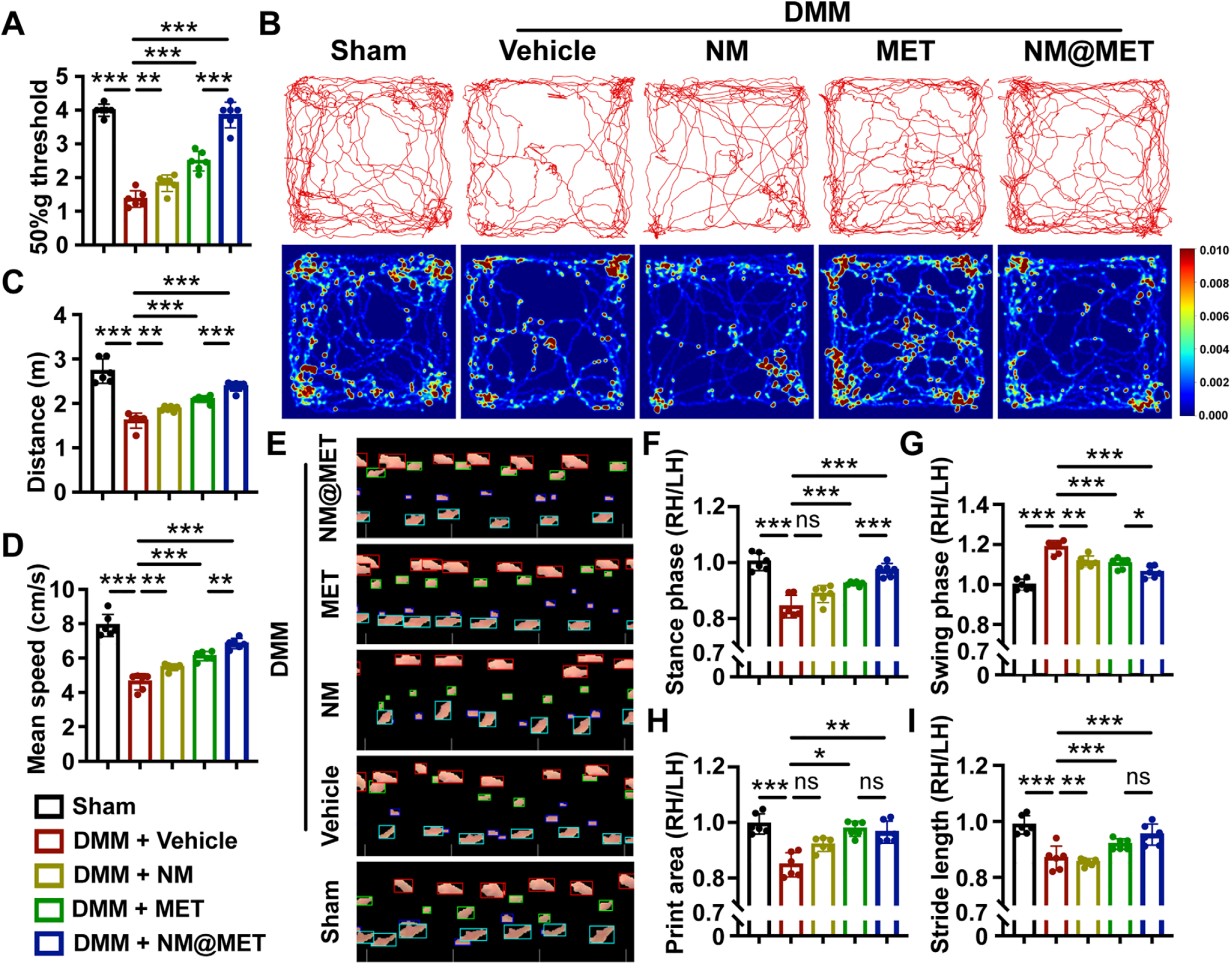
(A) Mechanical sensitivity was assessed by testing the thresholds of paw contraction using von Frey fibers. (B) Representative trajectory plots and heat map show that the spontaneous activity of mice after DMM surgery decreases in the open field test. Changes in spontaneous activity, including (C) distance, and (D) mean speed. (E) Representative pictures of the footprints of each group. NM@MET treatment group increased (F) relative stance phase, (H) print area, and (I) stride length, and shortened (G) relative swing phase in DMM mice. n = 6. ^*^ for *p* < 0.05, ^**^ for *p* < 0.01, ^***^ for *p* < 0.001, and ns > 0.05 respectively.

is home to nociceptors, which are specialized pain‐sensing neurons. Nociceptive neurons send two axons: one to the peripheral areas and joints, and another to transmit a pain signal to the spinal cord's dorsal horn [58]. The open field test swiftly evaluates overall locomotor activity by recording mouse movement trajectories in semi‐dark and silent conditions and providing an objective readout (Figure 8B). The distance and mean speed as expected, significantly decreased following DMM surgery compared to the sham group. This change was counteracted by the treatments (Figure 8C,D). In comparison to NM and MET, the NM@MET intervention demonstrated superior efficacy in restoring the exercise levels of mice. Additionally, we substantiated the pain and gait circumstances by footprint studies (Figure 8E). In the DMM group, a shorter stance phase and a significantly longer duration of swing phase were observed compared to the sham group. The NM@MET group exhibited an increased stance phase, a decreased swing phase, and improved the print area and stride length. (Figure 8F–I). These results indicated that NM@MET prominently reduced the pain and inhibited the mechanical allodynia of DMM mice.

## 3 Conclusion

In summary, we successfully developed ROS‐responsive nanomotors (AuNP@Pt‐PMO) with SOD/CAT activity. Nanomotor‐mediated sustained‐release of metformin has remarkable anti‐inflammatory and ROS scavenging properties in vitro. NM exhibited a combined effect with the loaded metformin. ROS‐motivated NM@MET augmented the penetration of metformin in murine cartilage and intensified the drug's effects, demonstrating a consistent, enduring, and substantial advantage over the treatment with metformin or nanomotors alone. Regarding the phenotypes of OA, NM@MET mitigates cartilage degradation, suppresses synovial M1 macrophage infiltration, diminishes synovitis, and curtails osteophyte production and subchondral sclerosis in the joint. In terms of pain management, NM and MET retain a strong combined effect. NM@MET diminishes the expression of molecules associated with DRG sensory conduction and alleviates pain in DMM mice. This nanomotor system represents a significant breakthrough in the field of intra‐articular drug delivery, offering a novel and efficient approach to cartilage protection and arthritis pain management.

## 4 Experimental Section/Methods

### 4.1 Materials

HAuCl_4_·3H_2_O, H_2_PtCl_6_·6H_2_O, L‐ascorbic acid (AA), sodium citrate, 1,2‐Bis (triethoxysilyl) ethane (BTEE), hexadecyl trimethyl ammonium bromide (CTAB), ammonia solution, and N, N‐dimethylimidodicarbonimidic diamide (metformin) were supplied by Aladdin. Ammonium phosphate dibasic was purchased from Sigma–Aldrich. Calcium nitrate tetrahydrate, ethanol, and hydrogen peroxide (H_2_O_2_, 10 wt.%) were obtained from Sinoreagent (Shanghai, China). All compounds were of analytical quality and were suitable for use without additional purification. DMEM medium was provided by Hyclone. Fetal bovine serum was obtained from Gibco. Dihydroethidium (DHE) was supplied by Applygen (Beijing, China). Calcein‐AM (CA) and propidium iodide (PI) were purchased from Solarbio (Beijing, China). Anti‐COL2A1, SOX9, MMP3, MMP13, ADAMTS5, iNOS, CD86, IL‐1β, FOS, NGF, TrkA, CGRP, and TRPV1 were acquired from Abcam (Cambridge, United Kingdom). Anti‐NRF2, KEAP1, HO1, GAPDH, and β‐ACTIN were supplied by Proteintech (Wuhan, China).

### 4.2 Characterization

The morphology and structure of AuNP, AuNP@Pt, and AuNP@Pt‐PMO were characterized by antransmission electron microscope (TEM, Tecnai F20, FEI, USA), and a field‐emission scanning electron microscope (Regulus8100, Hitachi, Japan). EDS mapping images of the nanomotors were observed by a high‐resolution transmission electron microscope (HRTEM, JEM‐2100F, JEOL, Japan). Zeta potentials were determined through DLS techniques, utilizing equipment from Brookhaven Instruments, USA.

### 4.3 Synthesis of AuNP

Introduce 250 mL of 0.02% HAuCl_4_·3H_2_O solution into a conical flask, heat to boiling, rapidly incorporate 5 mL of 1% sodium citrate while stirring vigorously, maintain heating and stirring for 15 min, cease heating, continue stirring for an additional 30 min, then halt and allow to cool naturally at room temperature. The samples were obtained through centrifugation at 10 000 × *g* and preserved at 4°C.

### 4.4 Synthesis of AuNP@Pt

160 mL of 0.375 mm AuNP solution and 70 mL of water were introduced into the flask, which was heated to 90°C and agitated for 10 min. 10 mL of 1 mm H_2_PtCl_6_ solution was combined with 80 mL of 4 mm AA solution using a 5 mL pipette. Following a 30‐min reaction, the mixture was cooled to room temperature, subjected to centrifugation at 10 000 × *g*, and stored at 4°C.

### 4.5 Synthesis of AuNP@Pt‐PMO Janus Nanomotors

30 mg CTAB and 0.5 mL ammonia solution were added to 10 mL of water, and the solution was stirred until it was clear. Then, 10 mL of 1 mg/mL AuNP@Pt was added, and the mixture was stirred for 30 min at room temperature to disperse evenly. 10 uL of BTEE was added drop by drop and stirred for 3 h. After collection by centrifugation at 10 000 × g, 20 mL of ethanol was added and sonicated for dispersion before stirring in a water bath at 60°C for 4 h. The samples were collected by centrifugation, dispersed in ethanol, washed three times in NH_4_NO_3_ ethanol solution (2 g L^−1^), dispersed in ethanol, and stored at 4°C.

### 4.6 Loading Efficiency of Metformin in Nanomotors

The maximum absorption of various metformin concentrations was assessed at 233 nm, and the calibration curve was constructed within the range of 100–2500 µm. The absorption and concentrations of metformin exhibited a linear correlation with drug concentration, represented by the equation y = 0.6468x – 0.0085. The nanomotors were submerged in a 100 mm metformin PBS solution and agitated at 37°C for 48 h. The supernatant was obtained post‐centrifugation, and the drug loading rate of the NM@MET (36 µm mg^−1^) was assessed.

### 4.7 Drug Release Behaviors

The initial MET concentration in NM@MET was adjusted to 36 mm. 1 mL NM@MET was incubated in a shaker (100 rpm) at 37°C. At scheduled time points, 100 µl supernatant was sampled after centrifugation and supplemented with 100 µl of fresh PBS. The procedure was conducted over 14 days. The standard curve was used to determine each sample's concentration.

### 4.8 Analysis of the Motion Behaviors of Nanomotors

H_2_O_2_‐driven nanomotors: The videos of the movements of the nanomotors in H_2_O_2_ solution with different concentrations were recorded by NanoSight NS300. The videos were taken for 60 s at a frame rate of ∼ 25 frames/s. These videos were analyzed using an MTrack2 plugin for ImageJ to record coordinates for each nanomotor. Using the formula MSD (Δt) = (xi(t+Δt)‐xi(t))^2^ (i = 2), where x is a two‐dimensional vector, i is an index to indicate x and y, and t is the time interval, the mean‐square‐displacement (MSD) of the Janus nanomotors was determined. The MSDs can be fitted to the following equation to determine D_eff_: D_eff_ = MSD/4Δt. The instantaneous velocity was calculated from the frame‐to‐frame displacement of each nanomotor. The average velocity was defined as the total trajectory length divided by the total tracking time, according to v = Σd_i_/ΣΔt, where d_i_ represents the displacement between two consecutive frames, and Δt represents the time interval between frames. Nanomotors dispersed in H_2_O without H_2_O_2_ were used as the Brownian motion control. Active propulsion was evaluated by comparing the average velocity, MSD profiles, and D_eff_ of nanomotors in H_2_O_2_‐containing H_2_O with those in the H_2_O_2_‐free H_2_O control. At least ten nanomotors were examined in each instance.

### 4.9 ICP‐MS Analysis

NM suspension was intra‐articularly injected into the knee joints of Sham and DMM mice at a dose of 10 µL per joint (1 mg/mL). At days 1, 3, 7, 14, and 21 after injection, whole knee joints were collected, weighed, and digested by microwave‐assisted acid digestion. The concentrations of Au, Pt, and Si were measured by ICP‐MS and normalized to tissue weight. The elemental levels were further expressed as relative values to evaluate the retention and clearance of NM in knee joints.

### 4.10 In Vivo Fluorescence Tracking of Cy5.5‐Labeled NM

Cy5.5‐labeled NM was prepared by reacting amino‐functionalized NM with sulfo‐Cy5.5 NHS ester in the dark, followed by repeated washing with PBS to remove free dye. Free Cy5.5 or Cy5.5‐NM was intra‐articularly injected into the knee joints of Sham and DMM mice. At 2 h, 8 h, 1, 2, 3, 7, and 14 d after injection, mice were anesthetized and imaged using an IVIS imaging system.

### 4.11 Cell Isolation and Culture

Knee cartilage was dissected from 5‐day‐old neonatal mice after carbon dioxide euthanasia. After cutting the articular cartilage into small pieces with scissors, it was digested with 0.25% trypsin for 30 min and 0.25% collagenase II in DMEM/F12 growth media for 6–8 h at 37°C. Chondrocytes were digested and subjected to centrifugation at 1500 rpm for 5 min. The supernatant was removed, and the resuspended cells were cultured in DMEM/F12 supplemented with 10% FBS at 37°C in an incubator. The macrophage cell line RAW264.7 and the sensory neuron‐like cell line ND7/23 were obtained from ATCC and grown in DMEM medium supplemented with 10% fetal bovine serum.

### 4.12 Chondrocyte‐Conditioned Medium Assay

Primary chondrocytes were stimulated with IL‐1β and treated with NM, MET, or NM@MET for 2 days. After treatment, the medium was replaced with fresh complete medium, and the cells were cultured for another 2 days to generate conditioned medium from pretreated chondrocytes. The collected conditioned medium was mixed with ND7/23 culture medium at a 1:1 ratio and used to stimulate ND7/23 cells for 2 days.

### 4.13 Cytotoxicity

The chondrocytes and RAW264.7 cells were cultured in 96‐well plates for 24 h. Thereafter, NM@MET was introduced to the cells and co‐incubated for 1, 3, and 5 days. The cells were subsequently washed with PBS three times and incubated with CCK‐8 reagent for 4 h. The cell viabilities were ultimately assessed at 450 nm using a microplate reader.

### 4.14 Dead and Living Staining

Chondrocytes and RAW264.7 cells were cultured in 24‐well plates for 24 h. NMs, MET, and NM@MET were co‐incubated with cells for another 24 h. Then, the cells were washed with PBS three times and stained with CA‐PI assays for 30 min. Finally, the stained cells were observed by a fluorescence inverted microscope.

### 4.15 Detection of ROS Production In Vitro

DHE was employed to assess the capacity of NMs, MET, and NM@MET to eliminate ROS in cells. Chondrocytes activated with H_2_O_2_ and RAW264.7 cells stimulated with LPS and IFN‐γ were incubated overnight in 24‐well plates and subsequently treated with 200 µl of NMs (1 mg mL^−1^), MET (3 mM), and NM@MET (1 mg mL^−1^), followed by incubation at 37°C for 4 h. Thereafter, the medium was eliminated, and DHE was introduced as a ROS fluorescent probe to the treated cell solution, followed by a 30‐min incubation. Following PBS washing, fluorescence images within the cells were examined using a fluorescence inverted microscope.

### 4.16 Western Blot Analysis

Murine chondrocytes and RAW264.7 cells were lysed in ice‐cold RIPA lysis buffer containing 1% protease and phosphatase inhibitors for 30 min. Cell lysates were centrifuged for 30 min at 4°C at 10,000 × g. Proteins of identical concentrations were subjected to electrophoresis on SDS‐PAGE (10%–15%) and subsequently transferred to PVDF membranes. Following the blocking of each membrane with 5% non‐fat milk in TBST for 1 h, primary antibodies were incubated overnight at 4°C on a shaker. A chemiluminescence kit in the ChemiDoc XRS System identified proteins following 1 h of incubation with horseradish peroxidase‐conjugated secondary antibody.

### 4.17 Quantitative Real‐Time Polymerase Chain Reaction

Total RNA was extracted from cells utilizing Trizol (Takara, Otsu, Shiga, Japan) following the manufacturer's guidelines, and cDNA was synthesized employing the HiScript II Q RT Supermix (Vazyme, Nanjing, China) for reverse transcription. Subsequently, qPCR was conducted utilizing ChamQ SYBR Color qPCR (Vazyme, Nanjing, China). All data were normalized to GAPDH.

### 4.18 RNA Sequencing

The total RNA of chondrocytes treated with NMs or NM@MET was extracted. RNA purity was assessed using NanoDrop One/OneC. The Qubit RNA HS Assay Kit quantified RNA. The Agilent 4200 TapeStation assessed RNA integrity. The TruSeq Stranded mRNA LT Sample Prep Kit (Illumina, San Diego, CA, USA) was used to build the libraries following the manufacturer's instructions. AMPure XP beads purified fragments during library generation. These libraries were sequenced on an Illumina PE150 to generate 125 bp/150 bp paired‐end reads. Beijing Novogene Technology Co., LTD sequenced and analyzed transcriptomes. The clean reads were aligned to the reference genome with Hisat2. The FPKM for each gene was computed with Cufflinks. HTSeq‐count counted gene reads. Expression analysis was done with the DESeq2 R package. Hierarchical cluster analysis showed gene expression patterns in different groups. R was used for hypergeometric distribution‐based KEGG pathway enrichment and GSEA analysis.

### 4.19 Animal Experiments

Ten‐week‐old C57BL/6 male mice (n = 30) purchased from Biont Company (Hubei, China) were used for our in vivo experiment. Mice were housed in the animal facility of Tongji Hospital. All animal procedures, including DMM surgery, intra‐articular injection, in vivo imaging, behavioral tests, and tissue collection, were approved by the Ethics Committee of Tongji Hospital (approval no. TJH‐202401003), and were conducted in accordance with the ARRIVE guidelines. Prior to the DMM surgery, the mice were anesthetized, and the right knee was sanitized and depilated. The joint capsule was incised, and the adipose tissue was excised to facilitate microscopic examination of the medial meniscus‐tibial ligament. The ligament was severed prior to suturing the joint capsule and dermal laceration. Mice were randomly divided into five groups: (i) Sham group: a sham operation involves cutting on the right side of the knee without damaging the joint capsule, (ii) DMM surgery group, (iii) NMs group, (iv) MET group, (v) NM@MET group. Mice in each group were intra‐articularly injected with 10 µl PBS, NMs (1 mg ml^−1^), MET (36 mM), and NM@MET (1 mg ml^−1^) weekly for 8 weeks post‐surgery.

### 4.20 Cartilage Permeability

FITC and FITC‐NMs were intra‐articularly injected into the knee joints of sham‐operated and DMM rats [59]. At 12 h post‐injection, knee joints were harvested, rinsed with PBS, embeded, and sectioned to a thickness of 10 µm. Sections were examined using fluorescence microscopy.

### 4.21 In Vivo ROS Luminescence Imaging

Images were captured with the IVIS Spectrum and analyzed utilizing Living Image 4.5.5 software from PerkinElmer. L‐012 probe (15 mg/kg, Sigma–Aldrich, USA) was periarticularly injected into the area around the OA knee. Mice were put in the imaging chamber after being given isoflurane anesthesia. Then, the luminescence image was captured.

### 4.22 Micro‐CT

MicroCT analysis was conducted on the knees of sham‐operated and DMM surgical mice utilizing a VivaCT 40 scanner at a resolution of 15 µm, with an x‐ray energy of 70 kVp and 112 µA (Scanco, Wangen‐Brüttisellen, Switzerland). In accordance with the manufacturer's guidelines, the three‐dimensional images were reconstructed. The periarticular osteophytes were selected as the region of interest (ROI). The ROI size was calculated blindly on all four knee condyles (0–3) on the medial and lateral aspects of the tibia and femur, and the average was utilized for statistical analysis.

### 4.23 Histological Analysis

Knee specimens were immersed in 10% EDTA for a duration of two weeks to achieve complete decalcification. The specimens were subsequently embedded in paraffin and sectioned into 5 µm slices, followed by H&E staining and Safranin O‐fast green staining. H&E staining was employed to assess synovial activation by quantifying synovial lining cell thickness on a scale of 0 to 3. The medial and lateral compartments of the joint were evaluated independently, and the total score from both compartments was reported, with a maximum possible score of 6. Cartilage degeneration was assessed in Safranin‐O/Fast Green‐stained sections utilizing a semi‐quantitative scoring system ranging from 0 to 6. Two independent graders, blinded to the conditions, evaluated each section, and the mean score was utilized for statistical analysis.

### 4.24 Immunohistochemistry and Immunofluorescence

Following deparaffinization and hydration, sections were placed in citrate buffer (10 mm citric acid, pH 6.0) overnight at 60°C to facilitate antigen unmasking. In immunohistochemistry, slices were stained with secondary antibodies coupled with horseradish peroxidase after being incubated with anti‐MMP3, anti‐MMP13, and anti‐Collagen II antibodies. The color was then developed using the secondary antibody and DAB technique. For immunofluorescence, knee sections were incubated with Anti‐F4/80, Anti‐iNOS, and Anti‐CD206 antibodies. DRG sections were incubated with Anti‐TRPV1, Anti‐CGRP, Anti‐NGF, and Anti‐TrkA antibodies. Sections were stained with secondary antibodies labeled with Alexa 488 or Alexa 594 following the incubation with primary antibodies. DAPI and NeuN (Boster, Wuhan, China) were used to identify the nucleus. The staining findings were quantified using ImageJ software.

### 4.25 Ex Vivo Culture of Human Cartilage Explants

Human normal cartilage and OA cartilage tissues were obtained from surgical specimens with written informed consent from the donors. The collection and use of human cartilage tissues were approved by the Institutional Ethics Committee of Tongji Hospital (approval no. TJ‐IRB20210127). Cartilage tissues were washed with sterile PBS and cut into explants of comparable size under sterile conditions. The explants were cultured in complete medium and treated with Vehicle, NM, MET, or NM@MET. After incubation, cartilage explants were fixed with 4% paraformaldehyde, embedded in paraffin, and sectioned.

### 4.26 Von Frey Fiber Test

Prior to the test, each mouse was housed in a cage of 4 cm × 3 cm × 7 cm with a wire mesh floor. The mice were then given 15 min to acclimate to a wire mesh grid, and the von Frey fiber was applied to the plantar surface of their right hind paw. The force applied to the mouse paw was progressively increased until paw withdrawal occurred, and the withdrawal threshold was recorded. The sensitivity of the reaction to pain increases with decreasing threshold for paw withdrawal.

### 4.27 Open Field Test (OFT)

DeepLabCut was used to evaluate the animals' exploratory behavior and spontaneous activity. Every mouse was positioned one after the other in an unlit, open, 50 by 50 cm square indoor area. In order to assess the mouse's relative activity, active time, distance, and mean speed, a camera was employed to track its activity trajectory in real time during a 5‐min period. Ten frames were extracted from four videos to annotate four anatomical regions of the mice (tip of the nose, left ear, right ear, and base of the tail). Thereafter, Python code was used to calculate the correlation data.

### 4.28 Gait Analysis

The CleverSys TreadScan apparatus was used to analyze mouse gait. The device is made up of a transparent belt treadmill with a treadmill chamber above it that is lit by outside lights. Mouse ventral movies were recorded by a high‐speed digital camera beneath the treadmill chamber. After a minute of unrestricted exploration, a 20‐s video was recorded to determine the location of the mice's footprints.

### 4.29 Statistical Analysis

Statistical analyses were performed using GraphPad Prism software (version 10). All experiments were independently repeated three times. Data are presented as mean ± SEM. Statistical significance between two groups was assessed using a two‐sided Student's t‐test, while comparisons among multiple groups were conducted using one‐way ANOVA followed by Tukey's multiple‐comparisons test.

## Author Contributions

**Meng Zheng**: methodology, investigation, writing – original draft. **Changyu Liu**: methodology. **Qin Xia**: investigation. **Arndt F. Schilling**: writing – review and editing. **Jiawei Jiang**: data curation. **Renpeng Peng**: data curation. **Zixing Shu**: data curation. **Tian Ma**: data curation. **Danni Luo**: formal analysis. **Yaoyu Zhang**: formal analysis. **Yibo Fan**: formal analysis. **Xuyuan Zhang**: formal analysis. **Song Li**: validation. **Kai Wang**: validation. **Wentao Ke**: validation. **Yuan Xiong**: visualization. **Yuanli Zhu**: visualization. **Fangzhi Mou**: conceptualization, writing – review and editing, supervision. **Jun Xiao**: conceptualization, supervision, funding acquisition. **Hao Zhu**: conceptualization, supervision, funding acquisition, writing – review and editing.

## Funding

This work was supported by the National Natural Science Foundation of China (Grant No. 82202673 and 82330075), the Natural Science Foundation of Hubei Province (Grant No. 2024AFB635 and 2022CFB274), the Key Research and Development Program of Hubei Province (Grant No. 2024BCB009 and 2024BCB036), the Medical Artificial Intelligence Fund of Tongji Hospital (Grant No. AI2024B06), the Interdisciplinary Research Program of Huazhong University of Science and Technology (Grant No. 2024JCYJ047), the National Key Research and Development Project (Grant No. 2021YFA1201400), and the Graduate Innovation Fund of Huazhong University of Science and Technology (Grant No. YCJJ20252404).

## Conflicts of Interest

The authors declare no conflicts of interest.

## Supporting information




**Supporting File**: advs76314‐sup‐0001‐SuppMat.docx.

## Data Availability

The data that support the findings of this study are available from the corresponding author upon reasonable request.
