# Supplementary material for: ROS‐Targeted Nanomotor Therapy in OA: Cartilage Protection and Pain Relief
Source: Adv Sci (Weinh). 2026 Jul 6:e76314. Online ahead of print. doi: 10.1002/advs.76314 (PMC13336049; doi:10.1002/advs.76314)
Supplement: Supplementary file 1 — Supporting File: advs76314‐sup‐0001‐SuppMat.docx. [file ADVS-9999-e76314-s001.docx]

Supporting Information

**ROS-Targeted Nanomotor Therapy in OA: Cartilage Protection and Pain Relief**

Meng Zheng^1^, Changyu Liu^1^, Qin Xia^1^, Arndt F. Schilling, Jiawei Jiang, Renpeng Peng, Zixing Shu, Tian Ma, Danni Luo, Yaoyu Zhang, Yibo Fan, Xuyuan Zhang, Song Li, Kai Wang, Wentao Ke, Yuan Xiong, Yuanli Zhu, Fangzhi Mou*, Jun Xiao*, Hao Zhu*


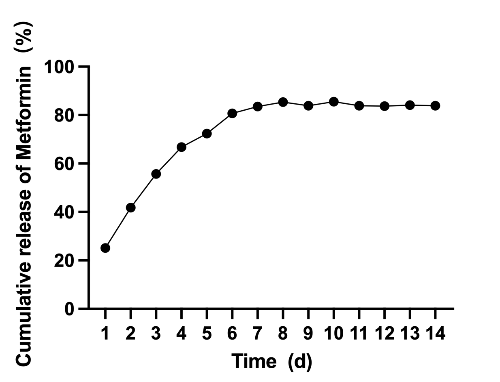


**Figure S1.** In vitro drug release curve of drug loading system.


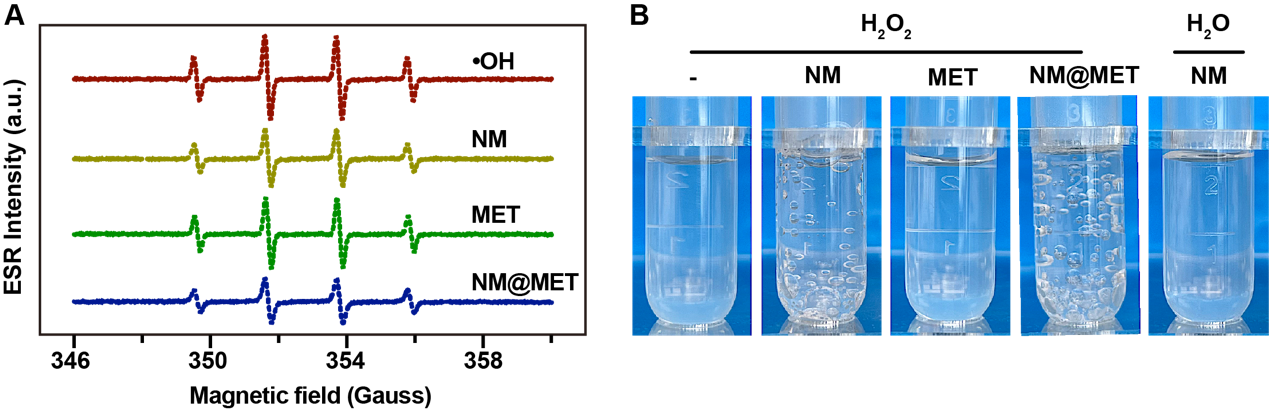


**Figure S2.** A) •OH scavenged by NM, MET, NM@MET ascertained by ESR. B) H_2_O_2_ was decomposed by nanomotors. The generated O_2_ bubbles were seen adsorbed on the inside of the tube wall. H_2_O was used as a negative control.


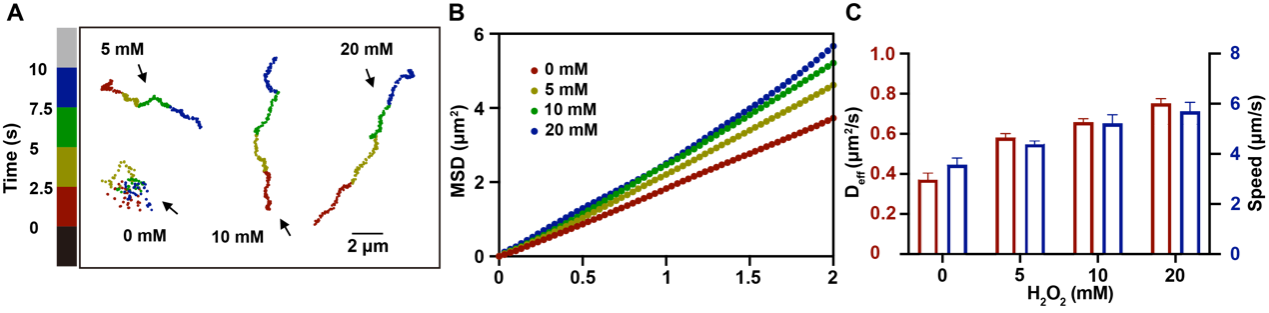


**Figure S3.** Motion behavior of nanomotors in DMEM medium with varying H_2_O_2_ concentrations (0, 5, 10, 20 mM). A) Time-lapse trajectories in 10 s, B) MSD, and C) D_eff_ and corresponding mean speeds under different H_2_O_2_ concentrations.


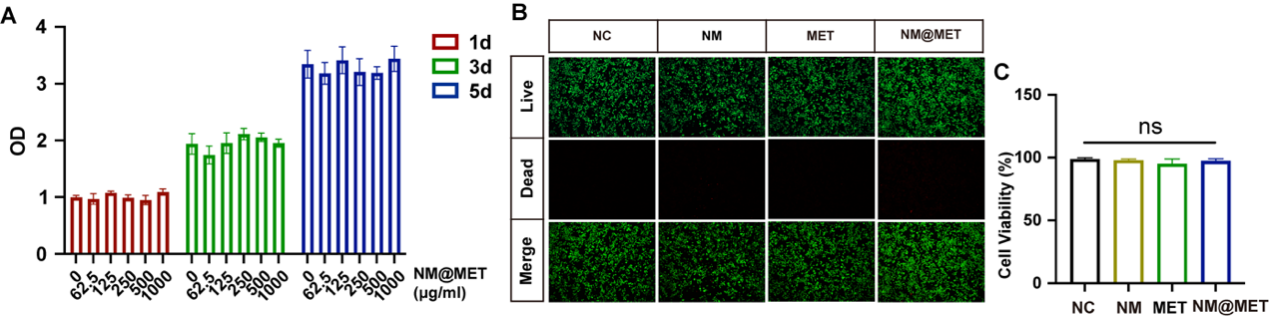


**Figure S4.** A) Cytotoxicity of NM@MET after 1, 3, 5d co-incubation with RAW264.7 cells. B) CA-PI staining images and D) quantification of dead and living cells treated with NM, MET and NM@MET.


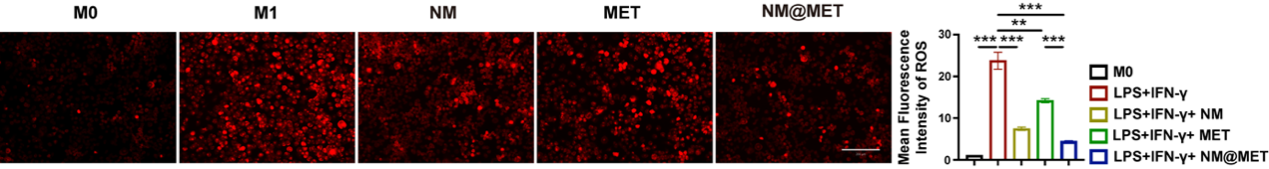


**Figure S5.** Fluorescence images and intensity of ROS probed in DHE in RAW264.7 cells.


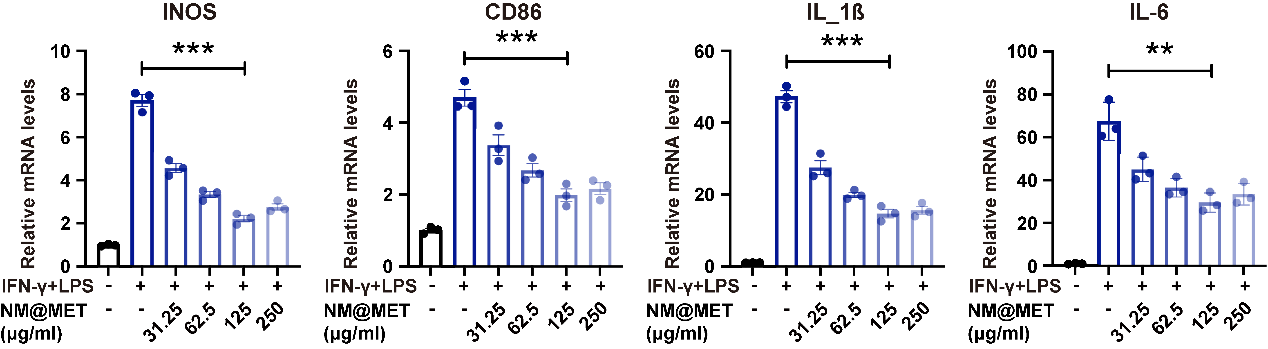


**Figure S6.** qPCR determined mRNA levels of INOS, CD86, IL-1ß, and IL-6 in RAW264.7 cells.


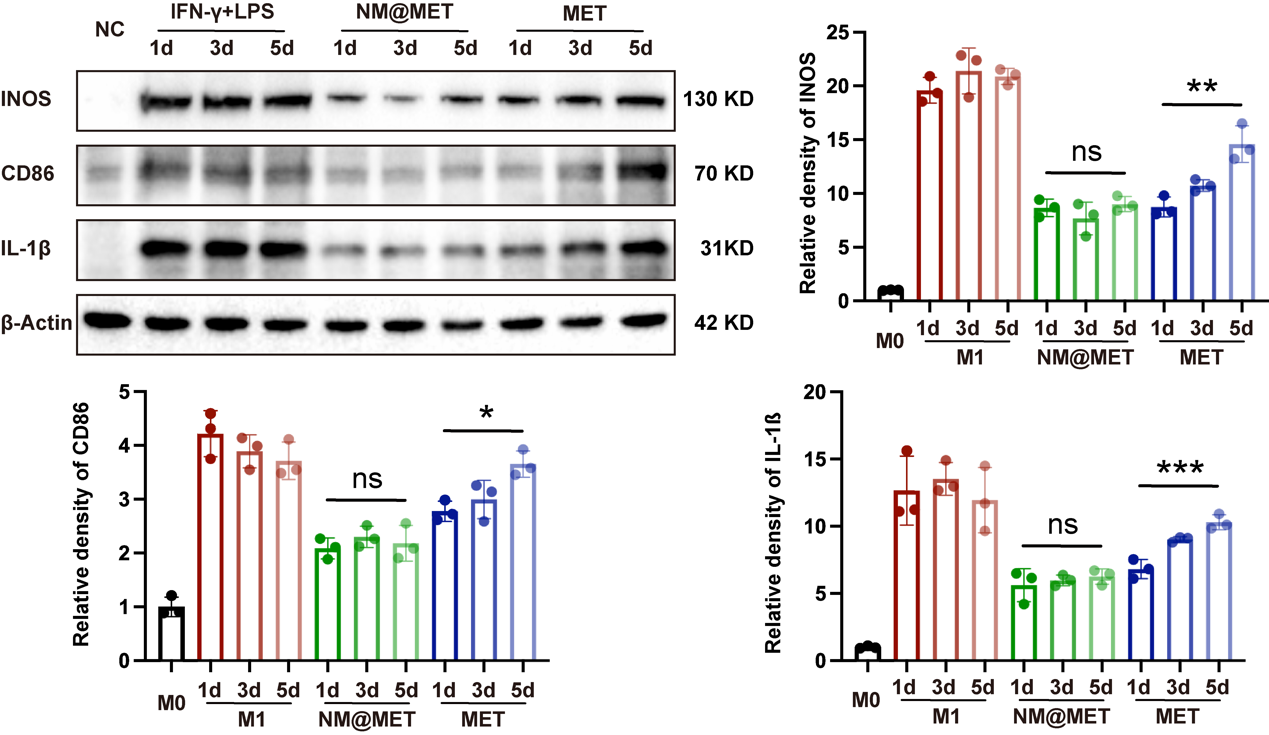


**Figure S7.** Representative western blot and quantification analysis of INOS, CD86, and IL-1ß. * is for p < 0.05, ** for p < 0.01, *** for p < 0.001, and ns > 0.05 respectively.


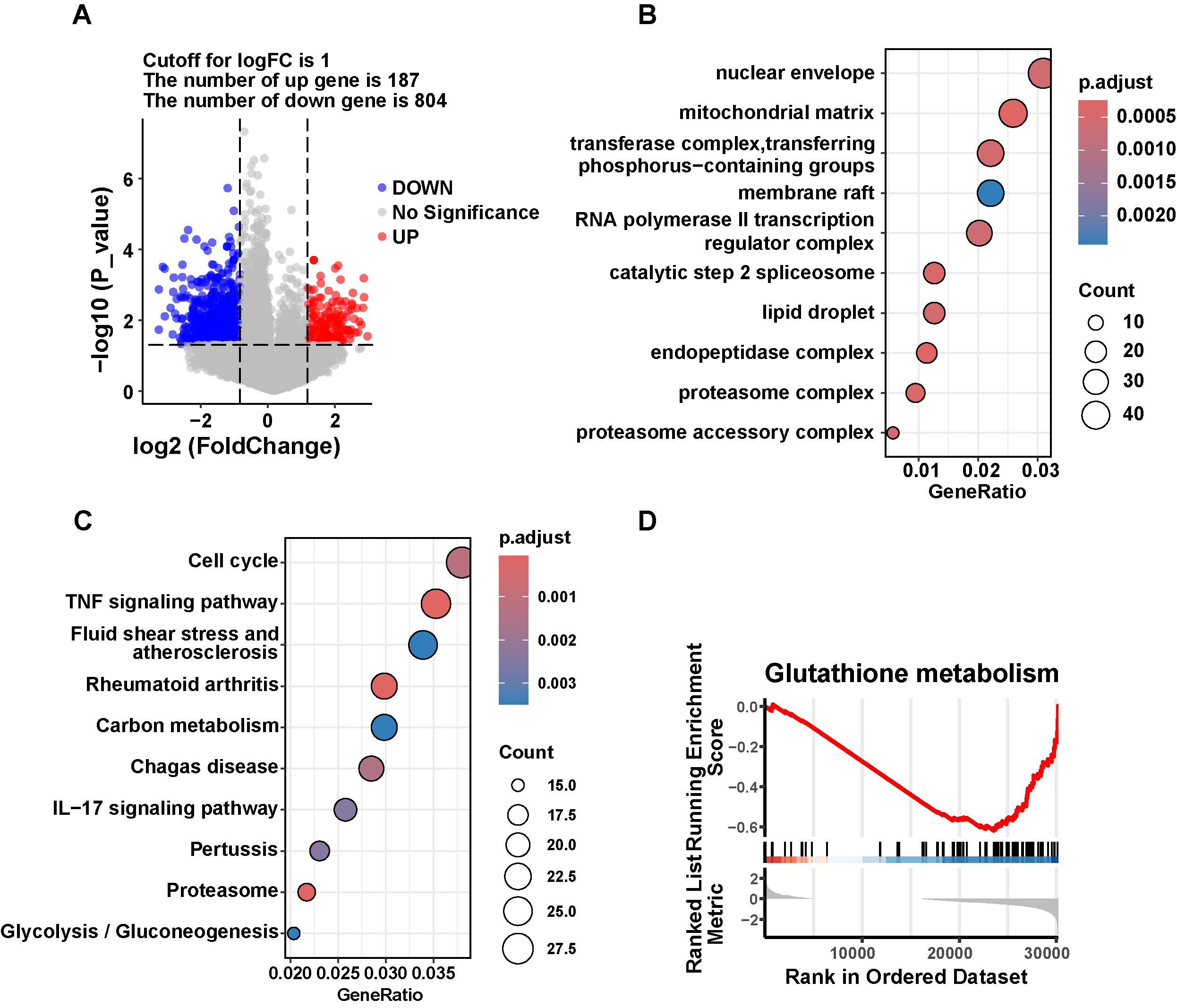


**Figure S8.** A) Volcanic plot of DEGs of NM@MET group_vs_IL group. B) GO analysis result of the DEGs of NM@MET group_vs_IL group. C) KEGG enrichment analysis result of the DEGs of the NM@MET group_vs_IL group. D) GSEA was used to identify the distribution of genes in glutathione metabolism pathway.


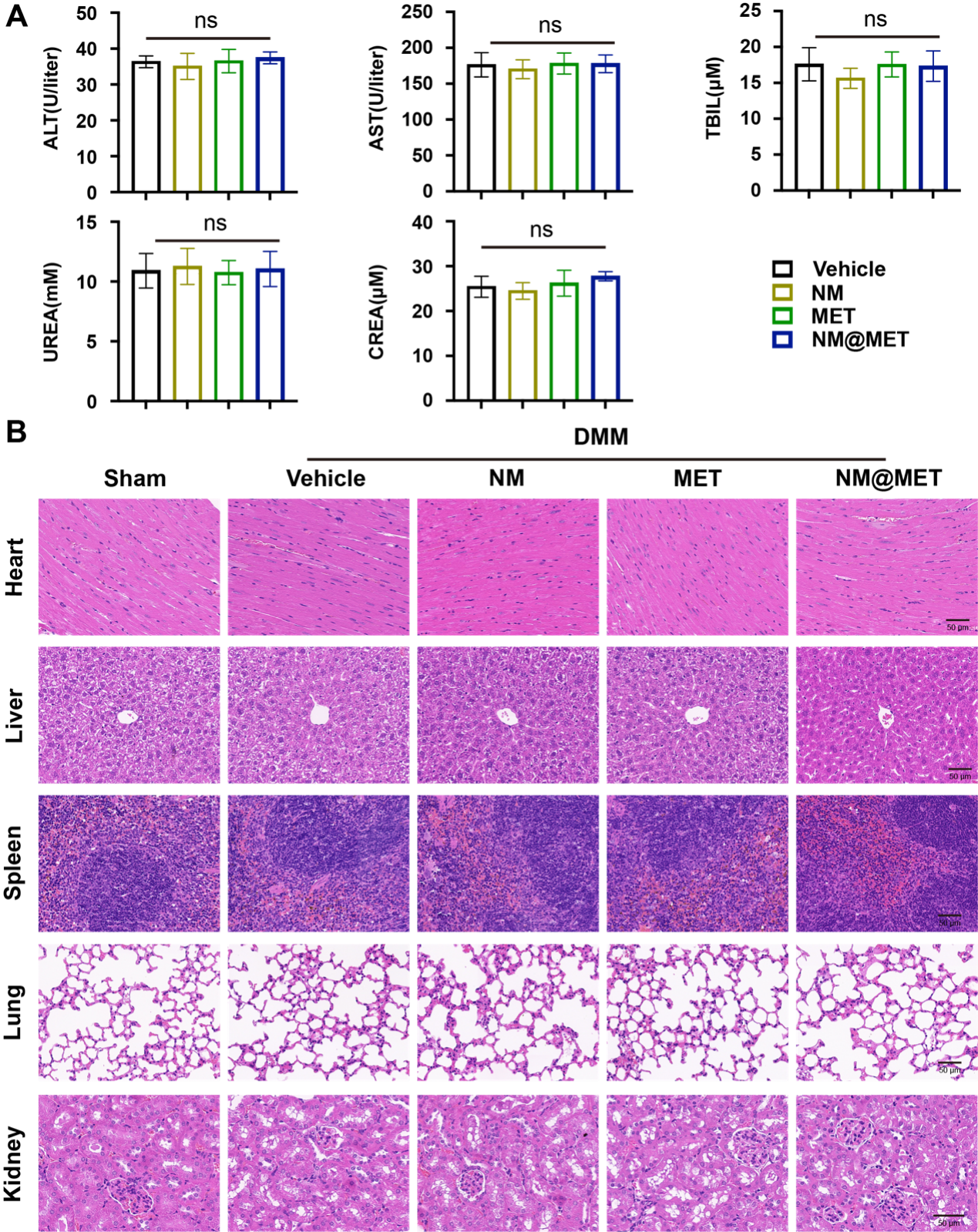


**Figure S9.** A) Serum biochemical analysis of mice after different treatments, including alanine aminotransferase (ALT), aspartate aminotransferase (AST), total bilirubin (TBIL), urea, and creatinine (CREA). B) Representative H&E staining images of major organs, including heart, liver, spleen, lung, and kidney, from Sham mice and DMM mice treated with Vehicle, NM, MET, or NM@MET. ns is for p > 0.05.


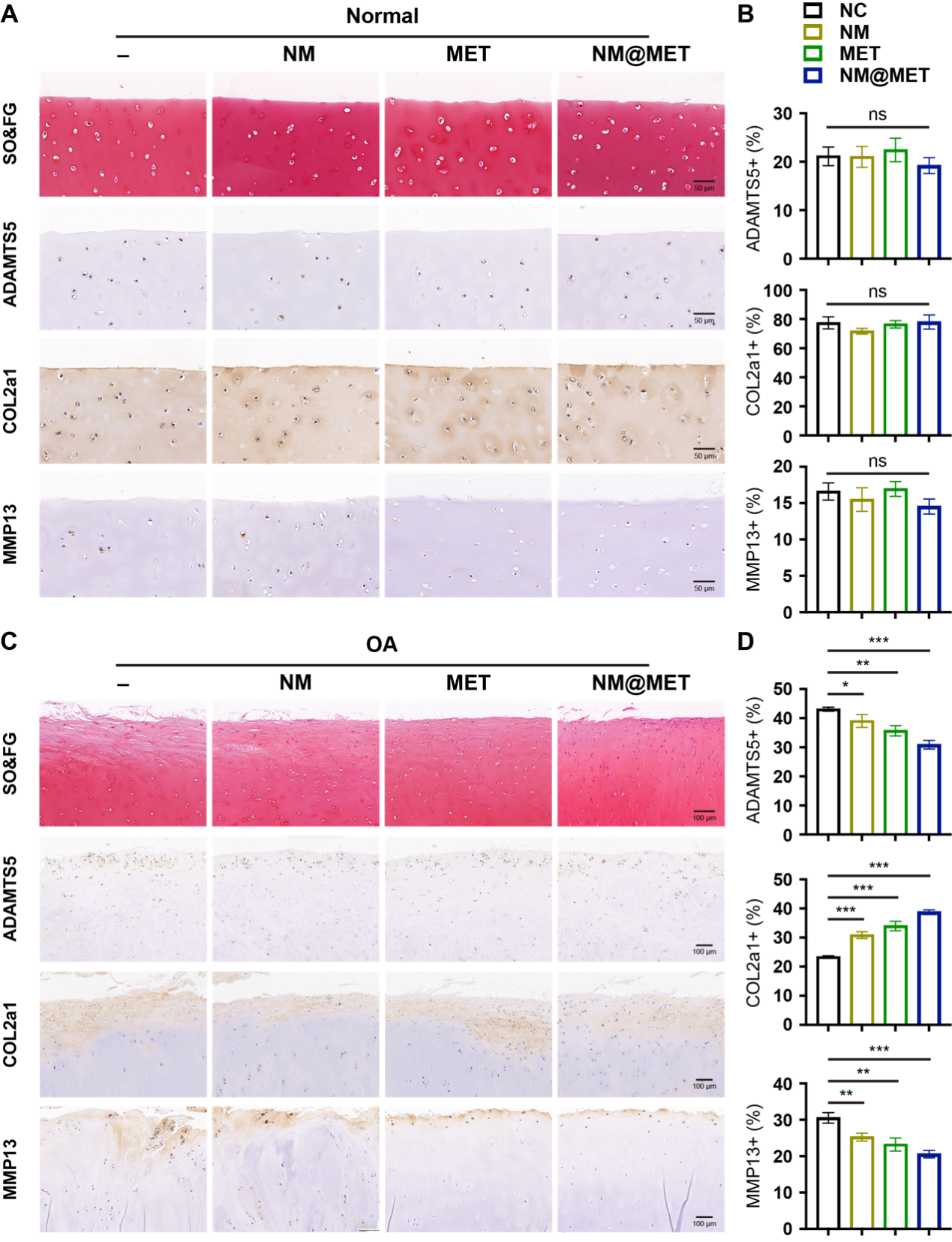


**Figure S10.** A) Representative Safranin O/Fast Green staining and immunohistochemical staining images of normal human cartilage explants after treatment with Vehicle, NM, MET, or NM@MET. B) Quantification of ADAMTS5, COL2a1 and MMP13 positive cells in cartilage. C) Representative Safranin O/Fast Green staining and immunohistochemical staining images and D) quantification of positive cells of human OA cartilage explants. Data are shown as mean ± SEM. ** is for p < 0.01, *** for p < 0.001, and ns > 0.05 respectively.


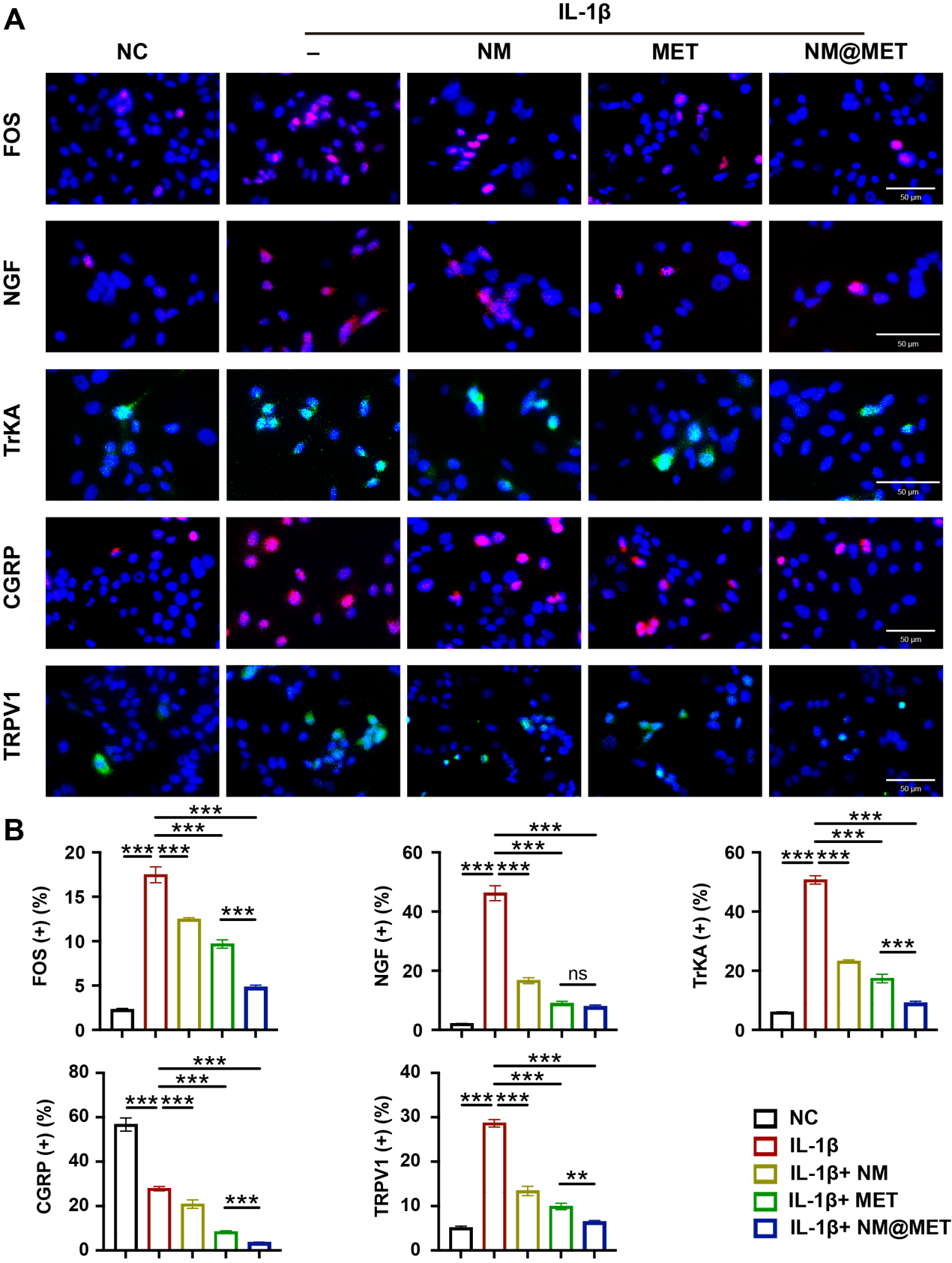


**Figure S11.** A) Representative immunofluorescence images of FOS, NGF, TrkA, CGRP, and TRPV1 in ND7/23 cells after stimulation with conditioned medium derived from chondrocytes subjected to different treatments. B) Quantification of FOS-, NGF-, TrkA-, CGRP-, and TRPV1-positive ND7/23 cells. Data are shown as mean ± SEM. ** is for p < 0.01, *** for p < 0.001, and ns > 0.05 respectively.
